# A Comparative Analysis of Dormancy and Germination of Arable Herb Seeds of Different Origins

**DOI:** 10.3390/plants15030485

**Published:** 2026-02-04

**Authors:** D. Gergő C. Á. Szemes, Luca Giuliano Bernardini, Leonid Rasran

**Affiliations:** 1 Institute of Botany, Department of Ecosystem Management, Climate and Biodiversity, BOKU University Vienna, Gregor-Mendel-Straße 33, 1180 Vienna, Austria; gergoe.szemes@boku.ac.at; 2 Institute of Soil Research, Department of Ecosystem Management, Climate and Biodiversity, BOKU University, 1180 Vienna, Austria

**Keywords:** weeds, conservation, Central Europe, ex situ, stratification, trait plasticity

## Abstract

Arable herbs rank among Europe’s most endangered species groups, calling for active conservation efforts to prevent their extinction. However, wild populations often cannot supply enough seeds for sustainable propagation, requiring seeds from botanical gardens or commercial producers instead. Yet, such ex situ plant populations can exhibit signs of significantly reduced long-term fitness, including increased germination rate and reduced dormancy. We studied eight arable herbs with respect to changes in their germination behavior under ex situ conservation and cultivation. We conducted germination experiments with seeds of different origins (wild, conservation, cultivation) in climate chambers. Germination tests were divided into two temperature regimes simulating sowing in autumn and spring. Our results show that four species (*Bupleurum rotundifolium*, *Cota tinctoria*, *Legousia speculum-veneris* and *Petrorhagia prolifera*) confirm the assumption that conservation and cultivation management reduce dormancy and increase germination rate. Two species (*Agrostemma githago* and *Silene noctiflora*) showed increased germination rate in non-wild populations, and another two (*Ranunculus arvensis* and *Scandix pecten-veneris*) behaved in the opposite way, showing increased dormancy and reduced germination rate in non-wild populations. These findings highlight the importance of preserving trait variability in ex situ populations and should be taken into account when planning restoration measures for segetal flora.

## 1. Introduction

Seed dormancy is a crucial life-cycle adaptation that evolved in diverse ways across different plant species. It ensures the survival of populations during adverse environmental conditions, particularly in short-lived and annual species, by stimulating germination only when the environmental conditions are suitable for seedling survival and plant growth until maturation. This ensures seed dispersal not only on a spatial but also a temporal scale [1]. However, seed germination and dormancy are governed by highly complex and sensitive biotic and abiotic interactions that vary significantly between species. At the species level, dormancy and germination rate are characterized by a high plasticity, and genes regulating dormancy and germination are subject to the strongest selection pressures in natural populations [1,2,3,4,5,6]. Therefore, seed dormancy and germination behavior are essential traits for the majority of arable herbs that fully depend on generative reproduction. Their adaptation to agricultural practices such as regular tillage, crop rotations, and multi-year cover crops for weed suppression, as well as weather fluctuations such as drought, makes the development of persistent seed banks a crucial part of their life strategy [7].

Seed dormancy, genetic backgrounds and physiological mechanisms of germination are well-studied areas [1,3,5]. However, the majority of the studies rely on research on a few model species such as *Arabidopsis thaliana* (L.) Heynh. and major crops [3]. Hence, there is a great lack of understanding of the complex pathways controlling dormancy and germination in most other species [3,5]. Consequently, there is a great need for further research regarding the processes involved in the natural balance of soil seed banks of wild plants, particularly in species of high conservational value [3], such as arable herbs.

Most arable herb species have been continuously present in Central Europe’s landscapes since the advent of agriculture. Their occurrence can be traced back as far as the late neolithic and bronze age, i.e., approximately 2000–4000 years B.C. For various species, primary non-anthropogenic habitats no longer exist in the European landscape or are not known. The evolution and co-evolution of these plants occurred within arable fields alongside crops [8,9,10]. As a result, arable herbs support many insect and bird species in agricultural landscapes by providing food and/or habitat. Thus, arable herbs contribute significantly to the biodiversity of the agricultural landscape and provide important ecosystem services [11,12]. However, since the middle of the 20th century, the intensification of agricultural practices, including application of herbicides and mineral fertilizers, simplified crop rotations and improved seed cleaning, as well as habitat fragmentation, have caused a dramatic decline in both species diversity and population density in the segetal flora. As a result, arable herbs are now listed among the most vulnerable species groups in Central Europe [11,13,14,15,16,17,18,19,20].

Given the endangered status of many arable herb species, numerous small- and large-scale conservation projects have been launched in different regions of Europe to protect existing remnant populations and counter the decline of arable herbs [8,21,22,23,24,25]. Such projects primarily focus on utilizing autochthonous material from wild populations, comprising genotypes adapted to environmental conditions of the respective area, to promote local populations or to establish new ones. However, wild populations are often not available in the surrounding area or cannot provide a sustainable supply of seeds for the growing number of restoration measures. Consequently, there is an increasing demand for seeds from external sources, such as ex situ conservation populations or seed producers [26,27]. Conservation cultures, in botanical gardens, for example, are often used as an alternate source to re-establish populations in the wild and are a globally accepted tool for species conservation [28,29,30].

Meanwhile, establishing flower strips from commercial seed mixtures has become common in European agriculture to promote biodiversity, often during fallow periods or as catch crops. Many such seed mixtures contain arable herbs such as *Agrostemma githago*, *Centaurea cyanus* or *Papaver rhoeas*, often sourced from horticulture. Their large, brightly coloured flowers make them appealing to the public, and their affordability has led to widespread use in ornamental landscaping [31,32,33,34].

The use of such (mass) cultivated seeds, however, raises concerns about genetic risks and ecological quality due to their non-local provenance [35,36,37]. The EU authorities recognized these risks and have enacted regulations to mitigate them in seed production and marketing [38,39]. Recent studies show that the relaxed selection pressures and altered environmental conditions in ex situ populations can cause noticeable differentiation from wild populations. Several reports indicate that plants kept under ex situ conditions exhibit losses of genetic diversity, e.g., due to inbreeding and outbreeding, genetic drift, and founder effects [35,40,41,42,43]. These losses can lead to reduced fitness and maladaptation to the natural environment and stress, as well as changes in phenology, phenotype and other important plant traits, including increased germination rates and reduced seed dormancy [44,45,46,47].

The use of seeds originating from non-wild populations, such as conservation or cultivation populations, for the conservation of arable herbs raises the question of whether reported risks of altered germination behaviors are also pertinent for this ecological group. For this purpose, we conducted germination experiments with a focus on germination behavior and dormancy in eight rare arable herb species in order to determine potential differences between seed of different origins.

Our research questions were formulated as follows:

1. Does horticultural, commercial or conservational cultivation of arable herbs affect their germination behavior and ability to form a persistent seed bank compared to wild plants?
2. Do arable herb species exhibit species-specific germination responses to the effects of conservation and cultivation management compared to wild populations?

Our hypotheses:

1. The ability to keep some of the viable seeds in the soil as a seed bank is greatly reduced or lost in seeds of arable herbs that have undergone horticultural conservation or agricultural cultivation, compared to seeds of wild origin.
2. Seeds of arable herbs that underwent horticultural conservation or agricultural cultivation germinate faster, and the short-term germination rate is higher, than seeds from wild populations.

## 2. Materials and Methods

### 2.1. Species Selection

Species selection was constrained by the rarity of arable herbs and limited availability from non-wild populations, such as ex situ conservation or cultivation populations. From an initial list of approximately 30 species, eight were selected based on (i) availability from at least one wild and one non-wild origin, and (ii) sufficiently large wild populations (≥500 individuals) for sustainable sampling. After careful investigation of available population data, the final species list comprised *Agrostemma githago* L. (*Ag*), *Bupleurum rotundifolium* L. (*Br*), *Cota tinctoria* (L.) J.Gay (*Ct*), *Legousia speculum-veneris* (L.) Chaix (*Ls*), *Petrorhagia prolifera* (L.) P.W.Ball & Heywood (*Pp*), *Ranunculus arvensis* L. (*Ra*), *Scandix pecten-veneris* L. (*Sp*) and *Silene noctiflora* L. (*Sn*). The nomenclature follows the Plants of the World Online Database (POWO) [48].

Most of the selected species are primarily winter annual therophytes, growing predominantly in warm and sunny sites on loamy and calcareous soils, belonging to the phytosociological alliance Caucalidion lappulae (R.Tx.1950) von Rochow 1951, within the order of Stellarietea mediae, with an exception being the perennial hemicryptophyte *Ct*, which is most commonly found in the phytosociological alliance of Convolvulo-Agropyrion repens Görs 1966 within the order of Agropyretalia repentis Oberd. et al. 1967. All selected species are insect-pollinated, with *Ag*, *Ls*, *Pp*, *Sp* and *Sn* also being capable of self-pollination [49,50,51,52,53,54].

### 2.2. Seed Sources and Sampling Site Characteristics

Before seed collection, source populations were categorized into origins as “wild”, “conservation” (cons.) or “cultivation” (cult.). Wild populations were defined as naturally occurring populations with a documented history under regular agricultural management and absence of direct human intervention with the aim of preservation of arable herb species. Conservation populations consisted of intentionally human-established populations (≥3 generations) originating from the wild source material and currently managed to support the conservation of the target herb species. Cultivated populations were defined as populations that were established ex situ specifically for seed production (incl. harvest and resowing the seeds of target species), predominantly using conventional agricultural or horticultural methods.

Sampling encompassed multiple sites in southwest Germany (Baden-Württemberg) and Austria (Lower Austria, Burgenland, Upper Austria). Population IDs were given based on country of origin, with A for Austria and G for Germany. German sampling sites were located in the rural district (Landkreis) ”Enzkreis”, known for multiple wild and autochthonous arable herb populations (G3–G7), with one cult. population in the first generation (G2) and one prominent conservation field (G1). This conservation field was established in 2009 from seeds of wild populations from the same district. It should be noted that it is a common agricultural field that is cultivated for crop production. Its management is very similar to conventional agriculture with additional measures for the conservation of the target species of arable herbs that have been established there. Conservation management in G1 includes no herbicide use, adapted crop rotations (e.g., exclusion of maize and sugar beet and preference for winter and summer cereals), broader sowing rows and reduced crop density, as well as adapted harvesting and tillage times to benefit species that complete their life cycle after crop harvest.

Austrian sampling sites included the arable herb cons. plots in the Botanical Garden of BOKU University (A1) and the botanical garden of the University of Vienna (A5), certified regional seed producer facilities (cult.) and several wild populations in agricultural and ruderal habitats (A3, A4, A6–A9).

Site conditions varied considerably, ranging from shallow loamy-sandy soils with very low to moderate carbonate content to deeply developed loamy soils with high carbonate and humus content. Annual precipitation ranged from 500–1000 mm across sampling locations. Wild populations occurred predominantly in organically managed fields, embankments, and ruderal habitats, while cultivated populations were maintained under conventional agricultural practices.

### 2.3. Seed Collection

Wild seeds were collected manually between June and August 2023 from 20–25 randomly selected individuals per population, following ENSCONET seed collection guidelines with sampling limited to at most 5% of a population’s individuals [55]. In case of critically endangered species (e.g., *Agrostemma githago*, *Scandix pecten-veneris*), a permission from the regional governmental nature conservation agency of Lower Austria was obtained. Ripe seeds were collected under dry weather conditions into labeled paper bags, subsequently cleaned of debris in the lab, and stored at room temperature for desiccation. Cultivated seeds were purchased from a certified regional producer in Germany (G8) and collected with permission directly from cultivation fields of an Austrian certified regional seed production company (A2).

### 2.4. Experimental Design

The experiments were conducted based on a mixed population approach. All seeds of each population were pooled to ensure balanced replication and adequate sample sizes. Per replicate, 20–200 seeds were used, depending on species and seed availability. The seeds of each replicate were placed in a petri dish on moist filter paper.

The germination tests consisted of 2 phases: (1) a 22-day pre-treatment, hereafter referred to as stratification phase, at 4–6 °C in a cold room (to simulate sowing between autumn and winter), followed by (2) a 21-day incubation phase in versatile environmental test chambers with a 12 h light/dark cycle at 15 °C/10 °C (simulating a sowing in spring-like conditions). The illumination inside the chamber consisted of 6 daylight fluorescent lamps of 3560 lumen (5200 K) each.

The seeds were evenly distributed between two treatments: (1) a stratified treatment, which passed through the stratification and incubation phase, and (2) an incubated treatment, which only passed through the incubation phase, and where seeds were stored in paper bags at room temperature during the stratification phase of the stratified treatment.

Five species (*Ag*, *Br*, *Ra*, *Sp*, *Sn*) received anti-fungal treatment with potassium permanganate solution (0.01–0.05% KMnO_4_) prior to germination to prevent mould interference. The remaining three species (*Ct*, *Ls*, *Pp*) did not receive anti-fungal treatment due to the small seed size, making it infeasible. Germination was monitored at 24-h intervals. Germinated seeds (radicle ≥ 2 mm) were counted and removed at each monitoring interval.

On the last day of the incubation phase, non-germinated seeds were tested for viability using 1% triphenyl tetrazolium chloride (TTC) solution, following established protocols [56,57]. Seeds were bisected and immersed in TTC solution for ≥8 h in darkness. Viable seeds showed red–pink discoloration due to dehydrogenase activity and were classified as dormant. Seeds showing no discoloration were classified as non-viable. Due to resource limitations, simultaneous viability testing of all replicates was not possible. The procedure was performed in batches of 6–20 replicates per day, depending on the rate at which the seeds of each species could be prepared for the application of the TTC solution. During this post-experimental phase, hereafter referred to as post phase, all replicates that had not yet been tested for viability remained in the germination chamber, and germination was continuously recorded in 2-day intervals. Seeds that germinated during the post-experimental phase were classified as dormant to allow consistent data analysis. On average, germination rates during this phase varied between 0–11% of the total viable seeds, depending on species and population, and can be viewed in detail in Appendix A, Table A2.

### 2.5. Statistical Analyses

Germination and dormancy percentages were calculated based on viable seeds only, excluding non-viable seeds from analyses to prevent skewed data representation. The Germination Rate Index (GRI) was used as a dependent variable allowing comparison of germination rate and completeness between groups. It was calculated according to Esechie (1994) [58,59] to assess germination rate and completeness: GRI = Σ(Gᵢ/dᵢ), where Gᵢ represents daily germination percentage and d_i_ the corresponding day number. “Origin” and “Treatment” were handled as factors.

The data was evaluated for variance homogeneity and normal distribution to test the ANOVA assumptions; non-parametric tests were applied in case of any violation. Additionally, a multiple linear regression was performed to determine which parameters significantly influenced the data of each species. For the analysis, a robust two-way ANOVA was used with the Yuens trimmed mean t-test post hoc to determine significant differences between groups (*p* < 0.05) [60].

Statistical analyses were conducted using R (version 4.4.2) [61] in combination with RStudio (version 2025.05.1 + 513) [62]. The ”caret” package [63] was used to implement ML algorithms. For robust statistics, the ”WSR2” package [64] was used. All data processing was performed using ”dplyr” [65], ”tidyr” [66] and ”tidyverse” [67]. Data visualization was performed using the package ”ggplot” [68].

## 3. Results

### 3.1. Agrostemma githago (Ag)

**Figure 1:**
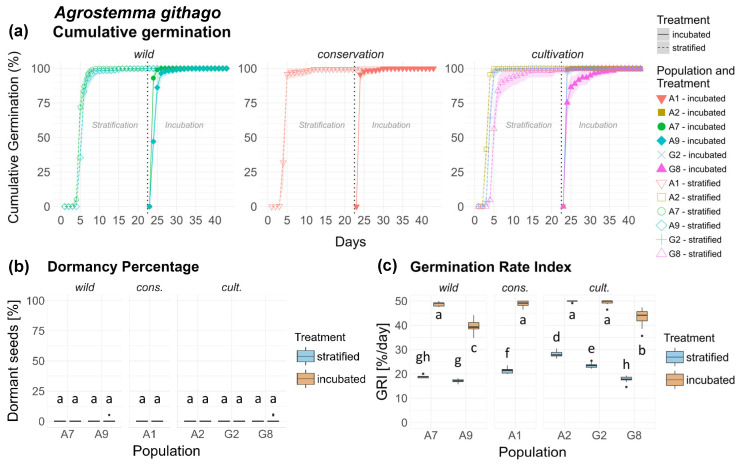
*Agrostemma githago*. (**a**) Cumulative germination (dotted vertical line indicates transfer of stratified seeds into the germination chamber), (**b**) Dormancy percentage and (**c**) GRI of the stratified and incubated treatment for each population. Significant differences (*p* < 0.05) between groups are marked with lower-case letters.

The germination of *Agrostemma githago* progressed very rapidly and homogeneously across all populations and origins. Nearly all seeds germinated within a few days after the seeds were placed in the petri dishes (Figure 1a). Only 2 out of a total of 4200 seeds of *Ag* were viable but didn’t germinate during the experiment (one seed in the incubated treatment of populations A9 and G8 each). Thus, no significant difference (Table 1) in dormancy could be found between treatments, populations and origins (Figure 1b).

**Figure A1:**
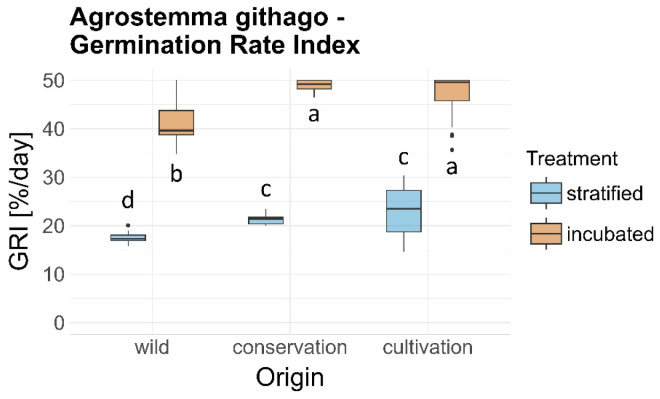
*Agrostemma githago*. GRI of the stratified and incubated treatment between origins. Significant differences (*p* < 0.05) between groups are marked with lower-case letters.

GRI varied significantly between populations (Figure 1c), with highly significant differences between treatments in all populations (Table 2). Population × treatment interaction was highly significant, whereas origin × treatment interaction showed much weaker effect. Concerning origins, populations of cons. and cult. exhibited a significantly, albeit only moderately, higher GRI compared to those of wild origin (Figure A1). Seeds of *Ag* are shown in Figure A7, Appendix B.

### 3.2. Bupleurum rotundifolium (Br)

**Figure 2:**
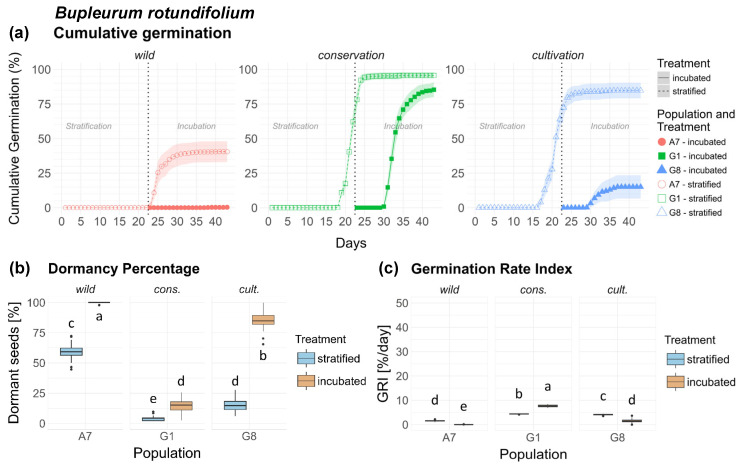
*Bupleurum rotundifolium*. (**a**) Cumulative germination (dotted vertical line indicates transfer of stratified seeds into the germination chamber), (**b**) Dormancy percentage and (**c**) GRI of the stratified and incubated treatment for each population. Significant differences (*p* < 0.05) between groups are marked with lower-case letters.

*Bupleurum rotundifolium* germination curves followed a sigmoidal pattern but very distinctly across populations (Figure 2a). In wild population A7, only seeds of the strat. treatment germinated, starting directly after transfer into the germination chamber and reaching a plateau at 41%. Cons. population G1 exhibited overall high germination in both treatments, whereas cult. population G8 showed high germination in the stratified treatment, and low germination in the inc. treatment. Differences in dormancy between populations and treatments were highly significant, as well as population × interaction (Table 1). Wild population A7 showed the highest dormancy among all populations (>50%) (Figure 2b), whereas cons. population G1 showed distinctly low dormancy in both treatments (<20%). Cult. population G8 showed opposing values between treatments, with very low dormancy in the strat. treatment (85%), and very high dormancy in the inc. treatment (15%). Concerning GRI, Br showed highly significant differences between populations/origins and treatments, as well as a highly significant population/origin × treatment interaction (Table 2). GRI was highest in cons. population G1, followed by G8 and lowest in A7 (Figure 2c). Seeds of *Br* are shown in Figure A8, Appendix B.

### 3.3. Cota tinctoria (Ct)

**Figure 3:**
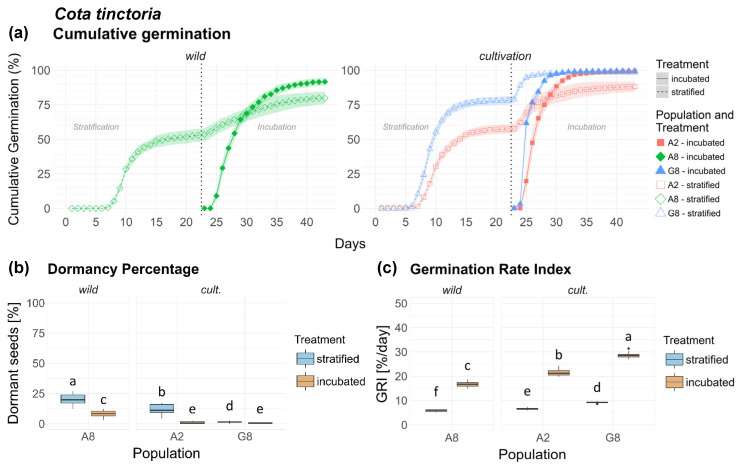
*Cota tinctoria*. (**a**) Cumulative germination (dotted vertical line indicates transfer of stratified seeds into the germination chamber), (**b**) Dormancy percentage and (**c**) GRI of the stratified and incubated treatment for each population. Significant differences (*p* < 0.05) between groups are marked with lower-case letters.

**Figure A2:**
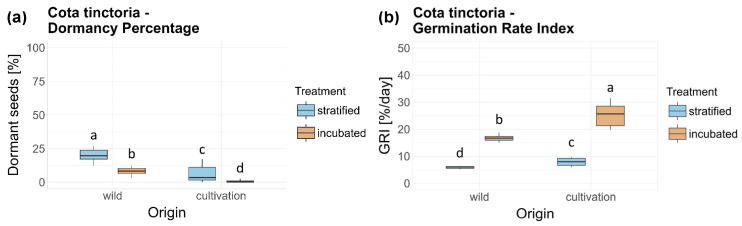
*Cota tinctoria*. (**a**) Dormancy percentage and (**b**) GRI of the stratified and incubated treatment between origins. Significant differences (*p* < 0.05) between groups are marked with lower-case letters.

All populations of *Cota tinctoria* displayed the same germination pattern independent from the origin. In the strat. treatment, germination followed a step-like curve with germination temporarily halting between phases. The inc. treatment, however, followed a sigmoidal germination curve with notably faster germination (Figure 3a). Differences in dormancy were moderate but highly significant between all populations and both origins and their treatments (Table 1). Wild population A8 displayed significantly higher dormancy in both treatments compared to both cult. populations A2 and G8, with G8 displaying the lowest dormancy of all populations (Figure 3b). Wild population A8 displayed significantly higher dormancy in both treatments compared to cult. populations A2 and G8, with G8 displaying the lowest dormancy of all populations. Population × treatment interaction was highly significant, while origin × treatment interaction was less pronounced (Figure A2a).

With respect to GRI, all comparisons were highly significant (Table 2), with cult. populations showing higher germination rate than wild ones. GRI was distinctly higher in the inc. treatment of all populations compared to the strat. treatment (Figure 3c). Treatment response was significantly higher in seeds of cult. origin compared to wild seeds (Figure A2b). Seeds of *Ct* are shown in Figure A9, Appendix B. 

### 3.4. Legousia speculum-veneris (Ls)

**Figure 4:**
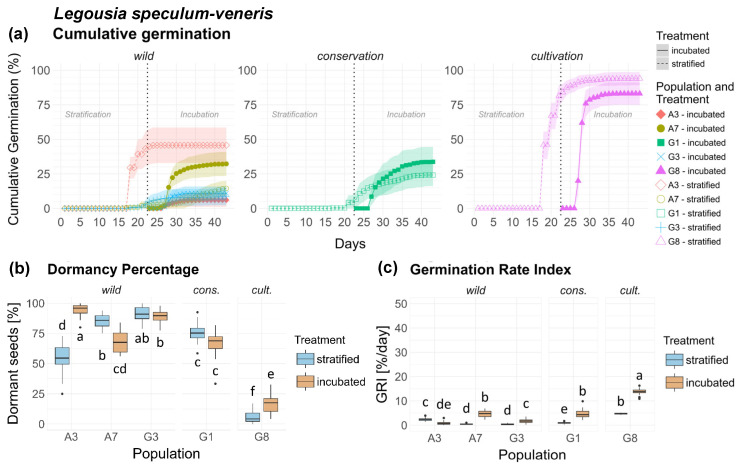
*Legousia speculum-veneris*. (**a**) Cumulative germination (dotted vertical line indicates transfer of stratified seeds into the germination chamber), (**b**) Dormancy percentage and (**c**) GRI of the stratified and incubated treatment for each population. Significant differences (*p* < 0.05) between groups are marked with lower-case letters.

*Legousia speculum-veneris* showed high variability across all populations and origins, including distinct variability between wild populations. Cult. population G8 exhibited particularly fast and complete germination compared to all other populations, whereas germination was more moderate in cons. population G1 (Figure 4a).

**Figure A3:**
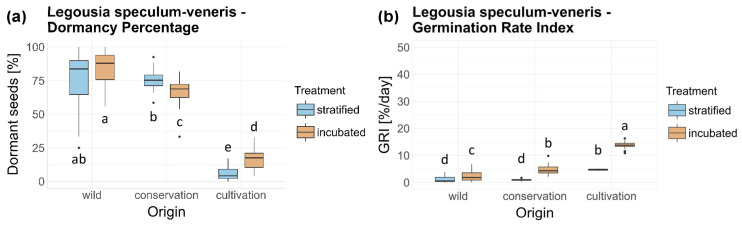
*Legousia speculum-veneris*. (**a**) Dormancy percentage and (**b**) GRI of the stratified and incubated treatment between origins. Significant differences (*p* < 0.05) between groups are marked with lower-case letters.

Dormancy was significantly higher in wild populations compared to cons. population G1 and cult. population G8 (Figure A3a), exceptions being the notably lower dormancy in the strat. treatment of A3 and the inc. treatment of A7 (Figure 4a). Treatment effect was not significant between origins, but highly significant between populations (Table 1), where three populations showed higher dormancy in the strat. treatment (A7, G3, G1), whereas two showed higher dormancy in the inc. treatment (A3, G8), but this was not significant between origins (Figure 4b). Population × treatment was highly significant, with the strongest effects in wild populations, while origin × treatment interaction was also highly significant, with effects being larger in non-wild populations.

GRI varied with high significance in *Ls* between populations and origins and their treatments (Table 2). Differences were moderate between wild and con. populations, while cult. population G8 displayed a distinctly high GRI in both treatments (Figure 4c). Population × treatment interaction was highly significant, as well as origin × treatment interaction, with treatment response being overall higher in non-wild populations (Figure A3b). Seeds of *Ls* are shown in Figure A10, Appendix B. 

### 3.5. Petrorhagia prolifera (Pp)

**Figure 5:**
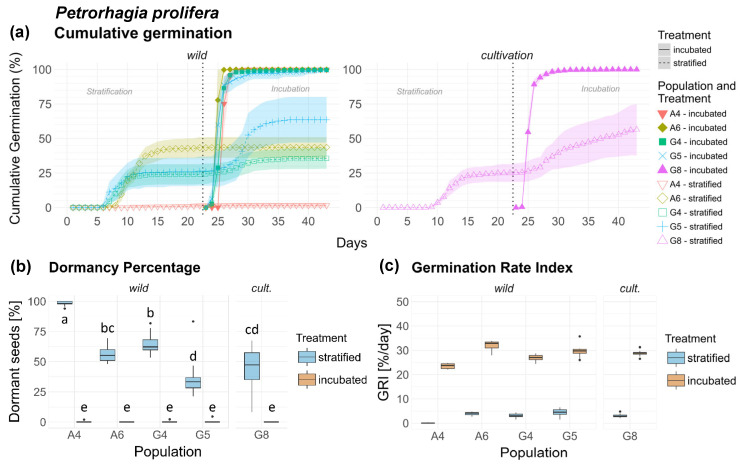
*Petrorhagia prolifera*. (**a**) Cumulative germination (dotted vertical line indicates transfer of stratified seeds into the germination chamber), (**b**) Dormancy percentage and (**c**) GRI of the stratified and incubated treatment for each population. Significant differences (*p* < 0.05) between groups are marked with lower-case letters.

The germination of *Petrorhagia prolifera* followed similar patterns across most populations. Seeds in the inc. treatment of all populations germinated rapidly and completely in a sigmoidal curve, whereas the strat. treatment germinated in a step-like pattern. Exceptions were the strat. treatment of wild population A4, which showed extremely low germination throughout the experiment (<3%), and A6, which halted germination in the strat. phase on day 17 (Figure 5a).

**Figure A4:**
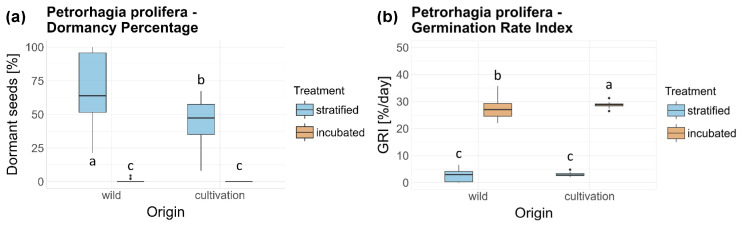
*Petrorhagia prolifera*. (**a**) Dormancy percentage and (**b**) GRI of the stratified and incubated treatment between origins. Significant differences (*p* < 0.05) between groups are marked with lower-case letters.

Dormancy differed with high significance between population and particularly between treatments Table 1), with highly significant differences in the strat. treatment of all populations, while the seeds in the inc. treatment germinated completely. In cult. population G8, dormancy levels ranged between those of G5 and A6, while dormancy was highest in wild population A4 (Figure 5b). The results show a highly significant variability between all populations, and particularly between wild ones. Between origins, dormancy was significantly higher in the wild than in the cult. origin (Figure A4a). Origin × interaction was also significant with a reduced treatment response in the cult. origin.

Differences in GRI were moderate, but significant between several populations (Table 2). Wild populations A4 and A6 showed the lowest and highest overall GRI, respectively. GRI of cult. population G8 ranged between those of wild populations G4 and G5 Figure 5c). Population × treatment interaction was highly significant, but small, and even smaller with respect to origin × treatment interaction (Figure A4b). Seeds of *Pp* are shown in Figure A11, Appendix B.

### 3.6. Ranunculus arvensis (Ra)

**Figure 6:**
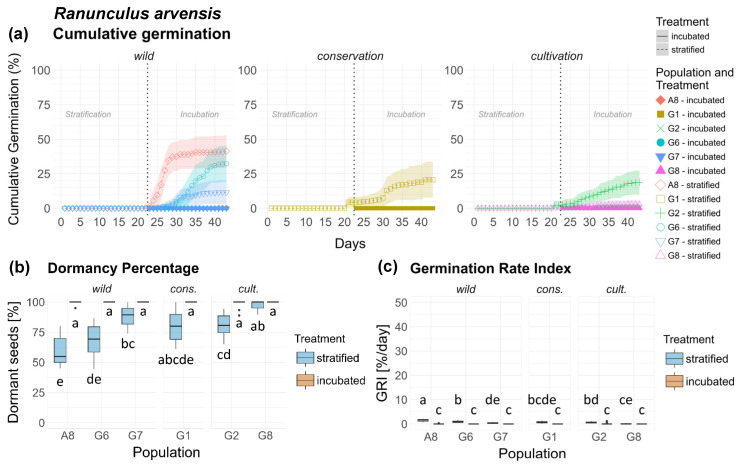
*Ranunculus arvensis*. (**a**) Cumulative germination (dotted vertical line indicates transfer of stratified seeds into the germination chamber), (**b**) Dormancy percentage and (**c**) GRI of the stratified and incubated treatment for each population. Significant differences (*p* < 0.05) between groups are marked with lower-case letters.

The seeds of *Ranunculus arvensis* exhibited a clear temperature-dependent dormancy, with almost no germination in the inc. treatment of all populations. In the strat. treatment germination remained negligible in the strat. phase and commenced mostly after transfer into the inc. phase (Figure 6a). Additionally, dormancy varied significantly (Table 1) between populations in the strat. treatment. Wild populations A8 and G6 exhibited significantly lower dormancy and the highest treatment response compared to all other populations (Figure 6b). Cult. population G8 demonstrated distinctly high dormancy without a significant treatment response. Dormancy levels and treatment responses between populations G7 (wild), G1 (cons.) and G2 (cult.) varied slightly but significantly. Between origins, differences in dormancy were highly significant, with a clear gradient of increasing dormancy and reduced treatment response from wild over cons. to cult. populations.

With respect to GRI, all analyses on population and origin level yielded highly significant results (Table 2). When viewed in absolute units, however, the differences were negligible (<2%/d) (Figure 6c). A seed of *Ra* is shown in Figure A12, Appendix B.

### 3.7. Scandix pecten-veneris (Sp)

**Figure 7:**
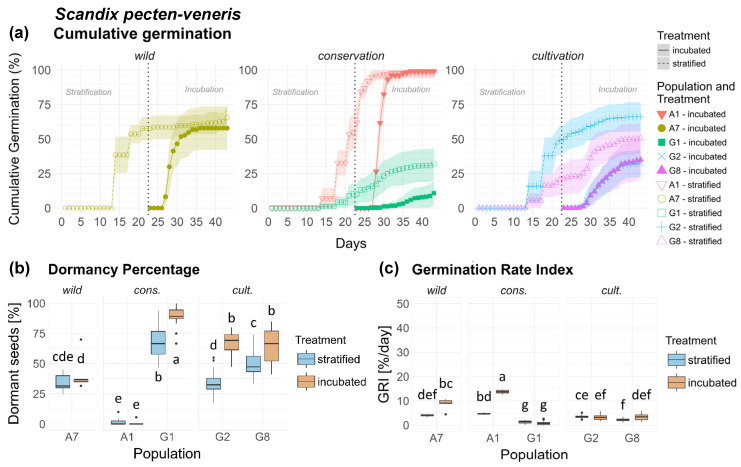
*Scandix pecten-veneris*. (**a**) Cumulative germination (dotted vertical line indicates transfer of stratified seeds into the germination chamber), (**b**) Dormancy percentage and (**c**) GRI of the stratified and incubated treatment for each population. Significant differences (*p* < 0.05) between groups are marked with lower-case letters.

**Figure A5:**
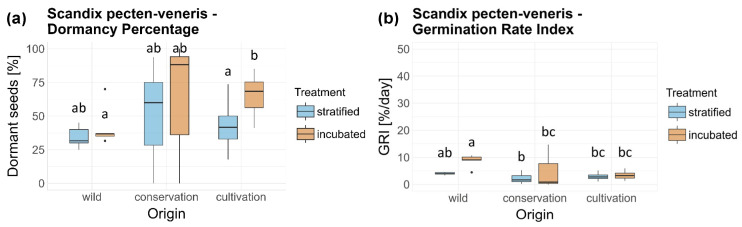
*Scandix pecten-veneris*. (**a**) Dormancy percentage and (**b**) GRI of the stratified and incubated treatment between origins. Significant differences (*p* < 0.05) between groups are marked with lower-case letters.

Germination behavior varied substantially among the five populations of *Scandix pecten-veneris* (Figure 7a), with highly significant differences in dormancy (Table 1) and GRI (Table 2) between populations and their treatments. Cons. population A1 demonstrated particularly high germination and low dormancy, while cons. population G1 showed the lowest germination capacity and highest dormancy, both significantly different from all other populations (Figure 7b). Wild population A7 exhibited intermediate dormancy and GRI levels, significantly different from all other populations. Cult. populations G2 and G8 displayed very similar germination patterns to each other and a particular overlap in the inc. treatment, with moderately high dormancy and low GRI. Stratified seeds generally showed more rapid and complete germination than incubated seeds of cons. population G1 and cult. populations G2 and G8. Treatment response varied among populations, as shown in Figure 7c. Overall, differences were much less significant between origins than between populations (Figure A5a,b).

Population × treatment interaction was highly significant in both dormancy and GRI. Wild population A7 and cult. population A1 demonstrated a comparatively high and significant treatment response in GRI, but not in dormancy. The remaining populations displayed negligible treatment response in GRI and a significant treatment response in dormancy, with higher dormancy in the inc. treatment. Seeds of *Sp* are shown in Figure A13, Appendix B.

### 3.8. Silene noctiflora (Sn)

**Figure 8:**
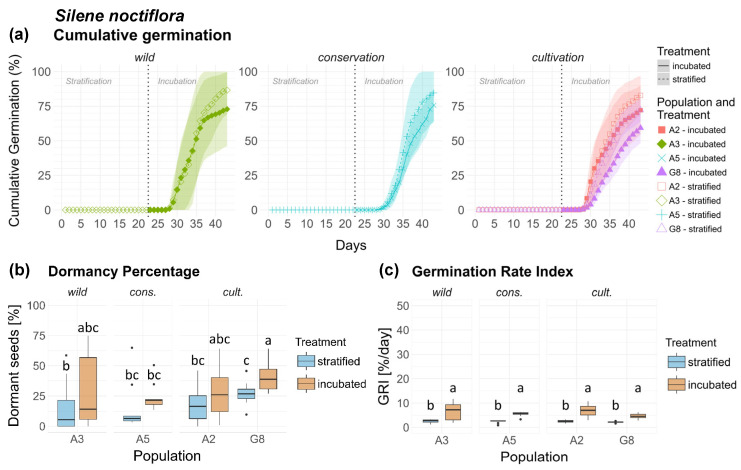
*Silene noctiflora*. (**a**) Cumulative germination (dotted vertical line indicates transfer of stratified seeds into the germination chamber), (**b**) Dormancy percentage and (**c**) GRI of the stratified and incubated treatment for each population. Significant differences (*p* < 0.05) between groups are marked with lower-case letters.

**Figure A6:**
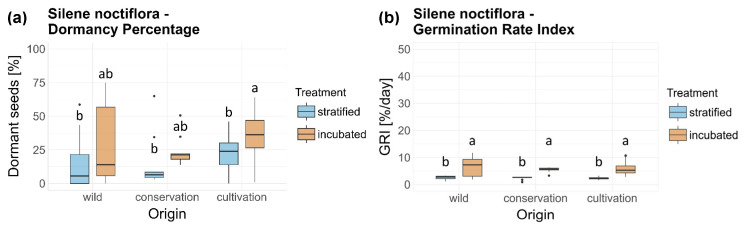
*Silene noctiflora*. (**a**) Dormancy percentage and (**b**) GRI of the stratified and incubated treatment between origins. Significant differences (*p* < 0.05) between groups are marked with lower-case letters.

**Figure A7:**
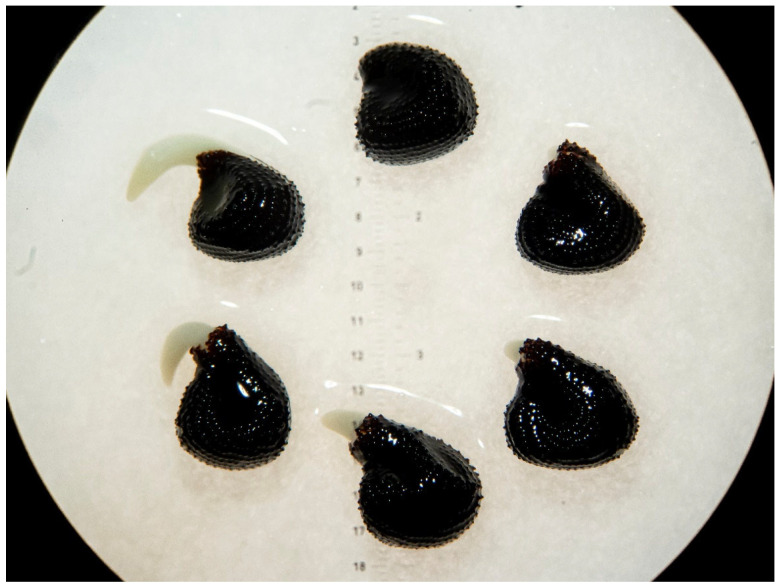
Seeds of *Agrostemma githago* shown in a clockwise germination sequence. Approximate seed diameter 4.5 mm.

**Figure A8:**
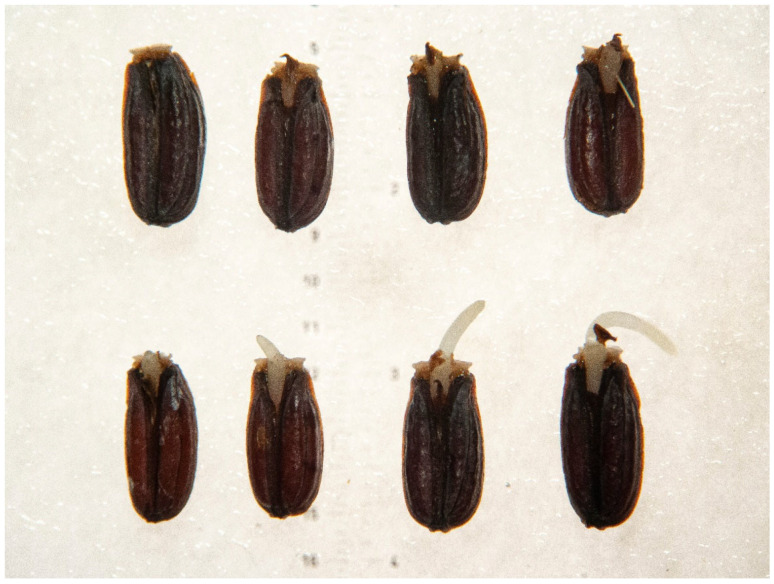
Seeds of *Bupleurum rotundifolium* shown in a germination sequence from top left to bottom right. Approximate seed length: 4 mm.

**Figure A9:**
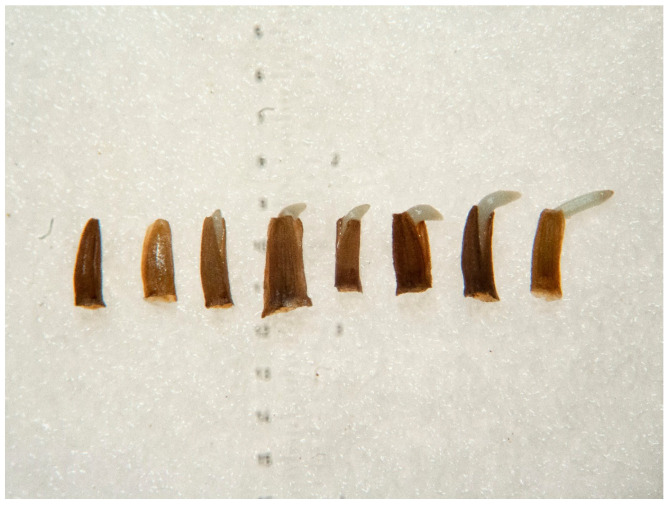
Seeds of Cota tinctoria shown in a germination sequence from left to right. Approximate seed length: 2.5 mm.

**Figure A10:**
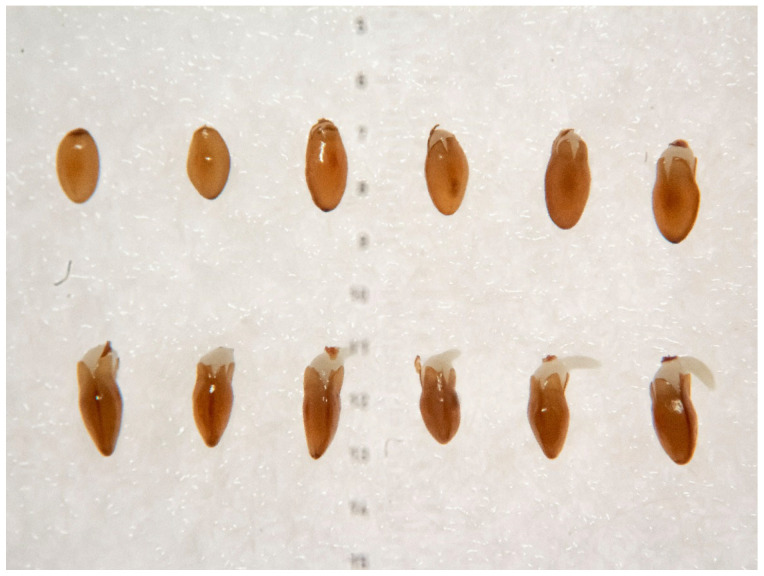
Seeds of Legousia speculum-veneris shown in a germination sequence from top left to bottom right. Approximate seed length: 1.5 mm.

**Figure A11:**
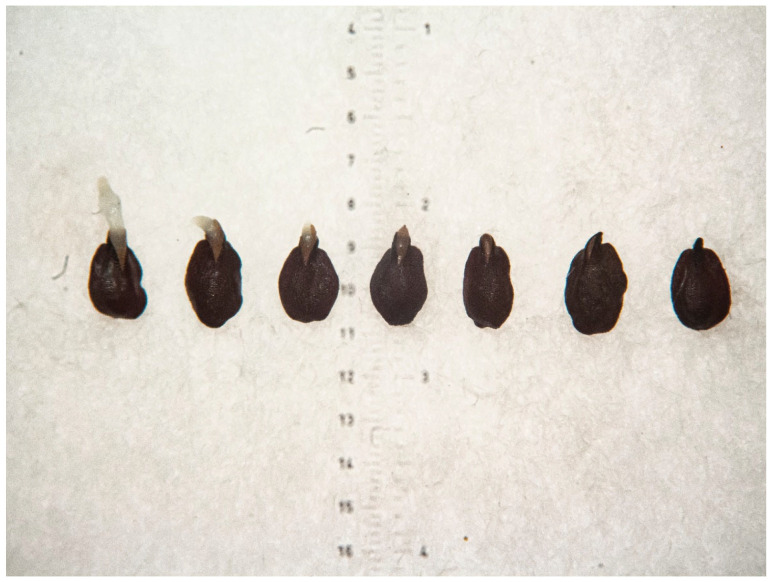
Seeds of Petrorhagia prolifera shown in a germination sequence from right to left. Approximate seed length: 2 mm.

**Figure A12:**
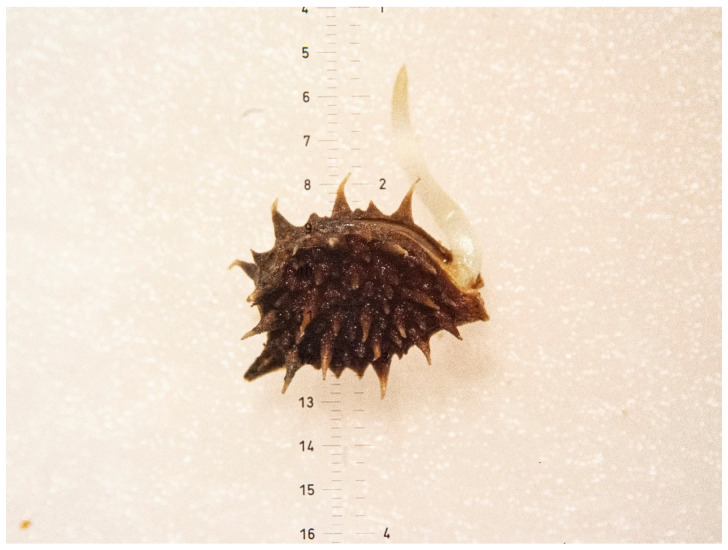
Germinating seed of Ranunculus arvensis. Approximate seed width: 6 mm.

**Figure A13:**
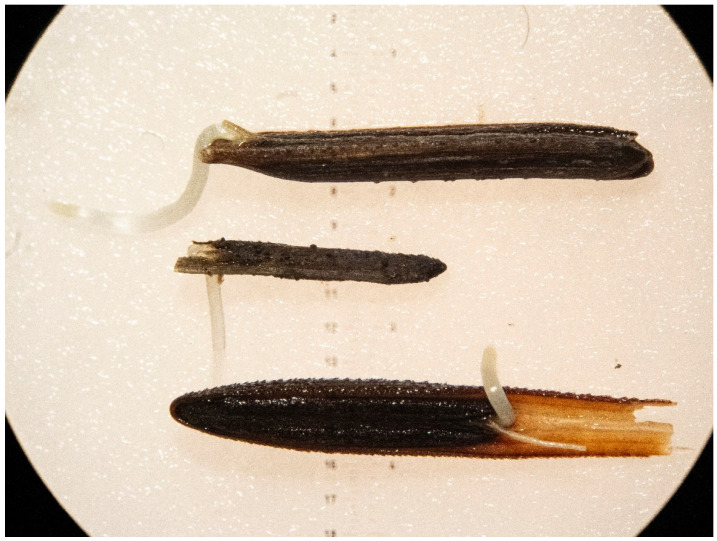
Germinating seeds of Scandix pecten-veneris. Approximate measurements of middle seed: 9 mm long, 2 mm wide.

**Figure A14:**
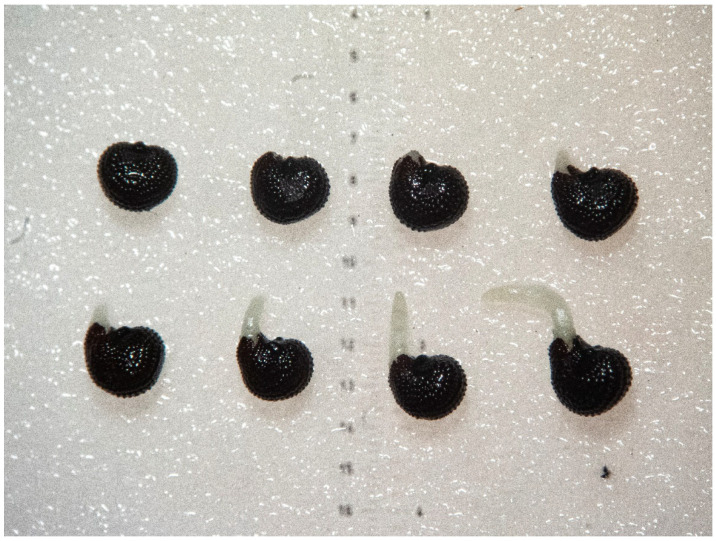
Seeds of Silene noctiflora shown in a germination sequence from top left to bottom right. Approximate seed diameter: 2 mm.

*Silene noctiflora* showed very similar germination patterns across all populations (Figure 8a). Low temperatures inhibited germination completely. Germination commenced 5–7 days after transfer into the germination chamber, with 1–2 days difference between populations. Total germination varied between 72–87% and 59–76% in the strat. and inc. treatment, respectively. Seed dormancy in *Sn* was significantly higher in cult. population G8 compared to all other populations (Figure 8b). Treatment effect was limited, but significant, with slightly higher dormancy in the inc. treatment than in the strat. treatment. Differences in dormancy between origins were significant, but relatively small, with a slight increase from wild over cons. to cult. populations (Figure A6a). Treatment effect on dormancy was significant across origins, while no significant interactions between population/origin × treatment were detected (Table 1). Concerning GRI, difference between populations and treatments were significant (Table 2), albeit marginal, with no significant differences in treatments across origins and treatment response between populations (Figure 8c) and origins (Figure A6b). Seeds of *Sn* are shown in Figure A14, Appendix B.

## 4. Discussion

We investigated the dormancy and germination behavior of eight rare arable herb species of five distinct phylogenetic groups representative of the segetal flora of Central Europe.

Existing germination data on three of the species are limited (*Ls*, *Ra*, *Sp*), whereas the five other species (*Ag*, *Br*, *Sn*, *Pp*) have been studied more extensively in different contexts [69,70,71,72,73,74,75]. Our results for the germination of *Ag*, *Br*, *Ct*, *Pp*, *Sp* and *Sn* are broadly consistent with the range of values reported in the Seed Information Database of the Society for Ecological Restoration [69], with notable exceptions being low germination rates in wild population A7 (0–37%) and the incubated treatment of cult. population G8 (10%) of *Br*. Our data for *Pp* are comparable only to incubated treatments due to missing data for temperatures below 15 °C. Also, results of *Ra*, *Sp* and *Sn* fall on the lower end of reported ranges. Previously published data on *Ls* from a study conducted in Germany report high germination rates (84%) under lab conditions, which is comparable to our results for cult. pop G8 (83–94%). However, the same study reported substantially lower germination rates (18.6 ± 2.4%) in open field conditions [76]. Overall, our results provide new information on three understudied species and validate findings for previously studied taxa.

The results of five out of eight studied species confirm our first hypothesis, that horticultural conservation and agricultural cultivation reduce dormancy. Species *Br*, *Ct* and *Ls* showed significantly reduced dormancy in both treatments of non-wild populations compared to wild populations. The results of *Pp* and *Sn* partially support this as well: *Pp* showed significant differences between seed origins in the strat. treatment, while the inc. treatment showed complete germination across all populations. *Sn* showed significant differences, but effects were smaller than in other the species. The results of the three species *Ag*, *Ra* and *Sp* refute the first hypothesis: *Ag* displayed no dormancy at all, whereas *Ra* and *Sp* showed increased dormancy in non-wild populations, opposing the hypothesis, (though weakly significant in *Sp*; Figure A5a).

Most species confirmed the second hypothesis: Five species (*Ag*, *Br*, *Ct*, *Ls*, *Pp*) showed significantly accelerated short-term germination (GRI) in non-wild populations. The remaining three species (*Ra*, *Sp*, *Sn*) refuted the hypothesis, with *Ra* and *Sn* showing no notable differences and *Sp* displaying significantly reduced germination rate in non-wild populations.

Overall, four species (*Br*, *Ct*, *Ls*, *Pp*) clearly support both hypotheses that conservation and cultivation reduce dormancy and increase germination rate. Two species show partial support of the hypotheses with accelerated germination only, while two species (*Ra*, *Sp*) exhibit opposite effects, with increased dormancy and reduced germination rate in non-wild populations.

### 4.1. Study Limitations

The discrepancies in germination and dormancy between the studied species is not surprising, as those traits are generally known to vary significantly between species [1,2,3,4,5,6]. Fresh and viable seeds are typically considered as dormant after 4 weeks without germination under favorable conditions (e.g., adequate moisture and temperature). However, dormancy classification is inherently context-dependent, as it is determined relative to the experimental or environmental conditions. Seeds classified as dormant under one set of conditions (e.g., temperatures, moisture, light regime) may germinate readily when conditions are altered [1,2,6,77]. Therefore, dormancy should be understood as a relative physiological state rather than an absolute trait, with determinations valid only within the specific environmental context. This also stresses the necessity to regard each species independently.

Another important aspect is the well-known effect of the applied methodology and test environment on germination outcomes. Germination rates of the same seed lot can differ substantially between laboratory and field conditions [44], underscoring the impact of experimental design on results. This is particularly relevant when ex situ material is tested under laboratory conditions prior to reintroduction, as several studies report laboratory germination rates up to 70% higher than subsequent germination in the field [76,78,79]. It is therefore to be expected that laboratory-obtained germination data overestimate true field germination rates of ex situ material. Exact causes for this shift remain unclear, but in our opinion, unintentional selection in ex situ populations may especially favor germination under laboratory test conditions. Moreover, it should be taken into account that germination response in annual species can vary substantially between years, depending on the weather conditions during the growth of maternal plants and maturation [80,81,82,83,84,85]. Besides local adaptations, phenology and fluctuations between years can affect germination dynamics. Such effects have already been observed in summer and winter annuals, where plants exhibited different dormancy levels, at different seed ripening periods and depending on life cycle strategies [86].

### 4.2. Differentiation Between Origins

Several studies report increased germination rate and reduced dormancy in non-wild populations compared to wild ones for various species [44,45,46,87,88,89,90]. These findings are often associated with negative effects on other traits, including reduced genetic diversity, lower pollinator attractiveness, phenological shifts and weakened fitness and stress response, as well as phenotypic changes (e.g., leaf length, plant height, seed size) [28,35,40,44,45,87,89,91,92]. A comprehensive study by Ensslin et al. [46] analyzing 72 species from 27 families found consistent dormancy loss in botanical garden material compared to wild counterparts, with short-lived species being significantly more affected. This suggests that dormancy loss is highly likely in arable herbs, which often have a particularly short life cycle. Additionally, studies on *Lotus conticultaus* L. demonstrate that such effects can appear after only one generation of cultivation [93], implying rapid adaptations in plants to ex situ conditions, causing traits to deviate from wild populations [28,41,94,95,96,97]. Moreover, several authors highlight that ex situ material used for reintroduction may negatively affect plant traits of wild populations through hybridization in subsequent generations [35,47,90]. This is, however, not a general pattern: While most species exhibit significant divergence between wild and non-wild populations, others show no significant differences [35,47,87,89]. This coincides with our observations.

### 4.3. Causes for Differentiation in Germination Behavior Between Origins

We expect that reasons for changes in germination behavior between wild and nonwild populations are attributable to several factors, including (i) unconscious anthropogenic selection during ex situ management, (ii) relaxed biotic and abiotic selection pressure (e.g., pests, inter-specific competition) in ex situ sites and (iii) varying environmental conditions (temperature, water and nutrient availability). This is especially relevant for cult. populations which exhibited the largest differences in dormancy compared to wild populations. However, causes are expected to be complex and multi-layered, including divergence due to founder effects and inbreeding in isolated populations, similarly to suggestions of previous authors [13,40,44,45,46,72,89]. Additionally, differences between populations may also reflect distinct ecotypes and thus natural variability within the species [1,2,3,4,5,6], particularly between distant populations from Austria and Germany, where, in recent years, annual precipitation and average yearly temperatures differed up to 100–200 mm and 1–2 °C, respectively [98,99]. In several cases (*Br*, *Ct*, *Ls*, *Pp*, *Sn*), we compared wild and non-wild populations that were not historically linked to each other according to our knowledge, suggesting that non-wild origins may also reflect ecotypes and not solely effects of seed origin.

### 4.4. Management Effects

Nevertheless, germination results can differ substantially also between populations that were assigned to the same origin based on our criteria. This is shown in the example of the two cons. populations of *Sp*, with A1 exhibiting the lowest and G1 the highest dormancy among all populations of *Sp*. This most likely reflects different management regimes of each site. While A1 is a botanical garden plot within the city of Vienna with warmer urban climate, removal of competitive weeds (e.g., *Cirsium arvense* (L.) Scop., *Coronilla varia* L.) and watering during summer drought, and absence of crops, G1 is an open field under agricultural management (see Section 2 p. 4), fully exposed to fluctuations in weather conditions and inter-specific competition. Additionally, G1 experiences high drought stress (also compared to other wild plots in the rural district) due to reduced soil water retention capacity, exposure and absence of irrigation. These factors likely explain why populations of site G1 can be expected to exhibit a germination behavior similar to that of wild populations across respective species (*Br*, *Ls*, *Ra*, *Sp*). This is supported by the results of *Ra*, where population G1 exhibits a dormancy range overlapping with wild populations G6 and G7 from the same region. Similarly, results for *Ls* in G1 remain within the observed variability of the wild populations, albeit somewhat lower than regional wild population G3. We therefore presume that G1 shows germination behavior similar to that of other regional populations for *Br* and *Sp*. Therefore, differences between wild population A7 and G1 possibly represent local ecotype adaptations. We thus assume that large-seeded species respond differently to selection pressures than small-seeded species. Under increased drought stress (regularly observed in G1), large-seeded species (*Ra*, *Sp*) could increase dormancy to await more favorable conditions, whereas small-seeded species (*Br*, *Ls*) tend to germinate early and rapidly to complete their life cycle before conditions become too harsh [100,101].

Although dormancy depth and germination are determined genetically to a high degree, environmental effects during seed ripening can significantly modify dormancy and germinability through maternal effects [3,4,28,102]. Thus, differing ex situ conditions could induce physiological changes in maternal tissues or embryos that alter germination behavior without genetic differentiation [1,35,103]. However, these explanations remain speculative and require further study.

While differentiation between wild and ex situ populations is evident, reasons why some species (*Ag*, *Br*, *Ct*, *Ls*, *Pp*, *Sn*) show reduced dormancy or accelerated germination, whereas others (*Ra*, *Sp*) show the opposite effect remain unclear. Our results also show considerable variability among wild populations in several species (*Ls*, *Pp*, *Ra*), raising the question whether differences between seed origins reflect current management effects or long-term adaptations of local wild source populations to traditional farming practices. This point is particularly relevant when wild seeds from Austrian populations were compared to German cultivated or conservation material (*Ag*, *Br*, *Ct*, *Sp*). Whether similar within-origin variability occurs in the species represented by only a single population per origin remains unknown. These uncertainties could not be addressed in the current study due to the restricted availability of matching wild and non-wild populations from the same region.

### 4.5. Seed Size as a Potential Predictor

Seed size is commonly used as a predictor for dormancy and germination behavior, as it is also directly linked to the amount of available resources and active substances to the seed’s embryo [100,104,105]. In our experiments, large-seeded species (*Ag*, *Ra*, *Sp* > 10 mg) exhibited origin effects opposing our expectations and clearly deviated from the patterns observed in the small-seeded species (*Ct*, *Ls*, *Pp* and *Sn* < 1 mg; *Br* ~2.5 mg).

*Ag* is well known for its ability to germinate across a wide range of environmental conditions, including temperatures of 5–35 °C, burial depths up to 4 cm, osmotic potentials up to 1.2 MPa and salinity up to 300 mM NaCl [71]. It has consistently been classified as highly germinable with a transient seed bank persisting one year or less [49,106]. Given the long-standing presence of *Ag* in Central European crop fields since the late neolithic [10,107], these traits most likely developed because its grain size resembles cereals such as spelt and wheat, causing their seeds to be regularly reaped and sown together over several millennia. This likely resulted in a convergent evolution of *Ag* with cultivated crops through unintentional anthropogenic selection. Similarly to secondary crops such as rye and oats, *Ag* lost its ability to form persistent seed banks and became highly dependent on regular re-sowing for its survival [70]. We propose this historic anthropogenic influence most likely explains the extremely homogeneous and complete germination across all populations in this study. However, the significant differences in GRI indicate that germination rate was less affected by historical factors. Historical selection possibly focused on consistently high germination upon sowing rather than on germination rate, allowing individual populations to diverge over time, covering different phenology of the main crops (winter vs. summer cereals).

Yet, the other two large-seeded species *Ra* and *Sp* present a completely different picture. Despite a comparatively long co-occurrence in Central European arable fields [107], they show clear dormancy in all populations, with higher dormancy in non-wild populations. While *Ra* and *Sp* were likely affected by the same anthropogenic influences as *Ag*, they do not exhibit a loss of dormancy. *Ra* and *Sp* differ in three key aspects from *Ag*: (i) Their seeds ripen considerably earlier (May–July) compared to *Ag* (June–September) [49]; (ii) They possess a distinctly rough diaspore morphology versus a comparatively smooth seed coat in *Ag*; and (iii) Their diaspores are exposed and easily detachable, whereas the seeds of *Ag* remain enclosed in tightly packed capsules opening upwards. These differences likely contributed to the preservation of dormancy-related traits in *Ra* and *Sp*. The spikes on the nuts of *Ra* as well as the rough surface and needle-like form of *Sp*-mericarps enable dispersal by animals (epizoochory) and humans (anthropochory) into other habitats with different selection pressures regarding dormancy. Beyond cereal fields, *Ra* and *Sp* naturally occur at ruderal sites, waysides, field margins, open meadows [108,109] and, in case of *Ra*, riparian mudbanks [110]. Consequently, seeds and pollen were likely regularly exchanged between habitats inside and outside of arable fields, preserving of dormancy-related traits as well as a wider natural plasticity thereof. This does, however, not explain increased dormancy in non-wild populations. We consider it unlikely that ex situ conditions specifically promote seed dormancy.

The small-seeded species (*Br*, *Ct*, *Ls*, *Pp*, *Sn*) also showed the ability to produce dormant seeds, with significantly higher dormancy in wild populations, corresponding to the findings of aforementioned studies and our expectations. In spite of their comparatively small seeds, their long-distance dispersal capacities are quite limited. We suppose that these species maintained their dormancy because their seeds fell directly onto the soil before or during harvest and were therefore less affected by repetitive re-sowing, in contrast to *Ag*.

### 4.6. An Outlook on Species Conservation

Long-term reintroduction success requires maintaining natural variability in dormancy, germinability and other traits, especially in conservation and cultivation scenarios [111]. This demands better understanding of processes causing divergence between wild and non-wild origins, with subsequent management adaptations to preserve natural trait variability in ex situ populations, particularly with respect to seed dormancy.

Consequently, the following recommendations can be given for species conservation projects: (i) Actively breaking dormancy just before sowing, as suggested by other authors [2]; (ii) Increased application of inter situ approaches for species conservation and reintroduction [91,112,113,114], similar to conservation population G1, as a method for better preservation of the natural spectrum of functional traits in settings closely resembling natural conditions; (iii) The assessment of reintroduction programs should focus more on mid- to long-term monitoring of establishment of target species, rather than solely on initial seedling emergence rates.

## 5. Conclusions

Our results show that several arable herb species are affected by dormancy loss (*Br*, *Ct*, *Ls*, *Pp*) and change germination behavior under conservation and cultivation management, while others remain indifferent (*Ag*, *Sn*) or even display increased dormancy (*Ra*, *Sp*). We also observed a notable variability in dormancy and germination rates between different populations, as well as between populations of the same origin in several species. Therefore, wild and non-wild populations may have developed specific dormancy and germination patterns, causing them to react in different ways and to varying degrees to ex situ cultivation. This highlights the need for further research of the mechanisms controlling dormancy and germination behavior in arable herb populations. Such knowledge is essential to better understand the processes involved in the natural balance of soil seed banks and their seasonal fluctuations in germinability, as well as how they are affected by anthropogenic influences.

Finally, we emphasize the importance of inter situ conservation approaches in environments closely resembling natural habitats, in order to mitigate adverse effects and maintain natural trait variability. This is particularly relevant for long-term re-establishment of target species, as it remains highly uncertain whether dormancy-related traits that have been lost during ex situ cultivation can be recovered through natural selection after re-introduction into nature.

## Figures and Tables

**Table 1:** Results of the 2-way trimmed means ANOVA for dormancy percentage on population level (left) and origin level (right). Agrostemma githago was not tested due to complete germination in all groups. Psi (ψ) values are given. Significant values are shown in **bold**. Level of significance is indicated as follows: *p* < 0.001 ***, *p* < 0.01 **, *p* < 0.05 *, *p* > 0.05 ns.

		Population	Origin
Species	Group	ψ	ψ
*Bupleurum rotundifolium*	Population|Origin	6655.20 ***	6655.20 ***
	Treatment	2428.19 ***	2428.19 ***
	Interaction	827.85 ***	827.85 ***
*Cota tinctoria*	Population|Origin	434.29 ***	150.83 ***
	Treatment	170.02 ***	83.75 ***
	Interaction	120.77 ***	11.81 **
*Legousia speculum-veneris*	Population|Origin	2861.92 ***	1819.34 ***
	Treatment	13.39 ***	3.52 ns
	Interaction	231.15 ***	30.55 ***
*Petrorhagia prolifera*	Population|Origin	1210.83 ***	11.16 **
	Treatment	2049.22 ***	316.15 ***
	Interaction	1210.83 ***	11.16 **
*Ranunculus arvensis*	Population|Origin	200.47 ***	30.48 ***
	Treatment	255.47 ***	92.15 ***
	Interaction	200.47 ***	30.48 ***
*Scandix pecten-veneris*	Population|Origin	2478.39 ***	54.79 ***
	Treatment	81.48 ***	9.24 **
	Interaction	125.88 ***	19.20 **
*Silene noctiflora*	Population|Origin	38.14 ***	17.84 **
	Treatment	13.50 ***	10.08 **
	Interaction	0.06 ns	0.03 ns

**Table 2:** Results of the 2-way trimmed means ANOVA for the Germination Rate Index (GRI) on population (left) and origin (right) level. Psi (ψ) values are given. Significant values are shown in **bold**. Level of significance is indicated as follows: *p* < 0.001 ***, *p* < 0.01 **, *p* < 0.05 *, *p* > 0.05 ns.

		Population	Origin
Species	Group	ψ	Ψ
*Agrostemma githago*	Population|Origin	1459.96 ***	11.02 **
	Treatment	16,472.97 ***	1443.60 ***
	Interaction	22.65 ***	6.58 **
*Bupleurum rotundifolium*	Population|Origin	5670.33 ***	5670.33 ***
	Treatment	18.27 ***	18.27 ***
	Interaction	1273.21 ***	1273.21 ***
*Cota tinctoria*	Population|Origin	1958.74 ***	111.67 ***
	Treatment	7020.82 ***	784.73 ***
	Interaction	608.15 ***	42.43 ***
*Legousia speculum-veneris*	Population|Origin	3318.05 ***	1797.86 ***
	Treatment	624.95 ***	578.22 ***
	Interaction	1269.85 ***	412.04 ***
*Petrorhagia prolifera*	Population|Origin	472.32 ***	10.19 **
	Treatment	9703.25 ***	6196.31 ***
	Interaction	52.75 ***	6.46 **
*Ranunculus arvensis*	Population|Origin	203.64 ***	111.64 ***
	Treatment	245.27 ***	68.90 ***
	Interaction	203.64 ***	111.64 ***
*Scandix pecten-veneris*	Population|Origin	1977.82 ***	9.46 **
	Treatment	281.03 ***	0.96 ns
	Interaction	721.82 ***	3.58 ns
*Silene noctiflora*	Population|Origin	20.81 **	37.35 ***
	Treatment	108.38 ***	1.32 ns
	Interaction	7.29 ns	2.71 ns

## Data Availability

The data presented in this study are available as Appendix A and Appendix B (see below). Further details can be obtained on request from the corresponding author.

## Abbreviations

The following abbreviations are used in this manuscript:

ENSCONET	European Native Seed Conservation Network
Ag	*Agrostemma githago*
Br	*Bupleurum rotundifolium*
Ct	*Cota tinctoria*
Ls	*Legousia speculum-veneris*
Pp	*Petrorhagia prolifera*
Ra	*Ranunculus arvensis*
Sn	*Silene noctiflora*
Sp	*Scandix pecten-veneris*
Cons.	Conservation origin
Cult.	Cultivation origin
GRI	Germination Rate Index
TTC	triphenyl tetrazolium chloride

## Appendix A

**Table A1:** Mean ($\bar{x}$) percentage and standard deviation of germinated, dormant and non-viable seeds of each treatment, population and species; sorted by origin. Origins are given as wild, conservation (cons.) and cultivated (cult.). Values were calculated based on sample size (*n*; seeds per Petri-dish) and are given as average across all replicates (r).

				Stratified Treatment			Incubated Treatment		
Species	Population	Origin	r	$\bar{x}$ Germination	$\bar{x}$ Dormancy	$\bar{x}$ Non-Viable	$\bar{x}$ Germination	$\bar{x}$ Dormancy	$\bar{x}$ Non-Viable
*Agrostemma githago* (*n* = 20)									
	A7	wild	5	100 ± 0%	0 ± 0%	0 ± 0%	100 ± 0%	0 ± 0%	0 ± 0%
	A9	wild	20	100 ± 0%	0 ± 0%	0 ± 0%	99 ± 2.6%	0.3 ± 1.1%	0.8 ± 1.8%
	A1	cons.	20	97.8 ± 3.8%	0 ± 0%	2.3 ± 3.8%	97.5 ± 3.4%	0 ± 0%	2.5 ± 3.4%
	A2	cult.	20	99.8 ± 1.1%	0 ± 0%	0.3 ± 1.1%	100 ± 0%	0 ± 0%	0 ± 0%
	D2	cult.	20	99 ± 2.1%	0 ± 0%	1 ± 2.1%	99.3 ± 1.8%	0 ± 0%	0.8 ± 1.8%
	G8	cult.	20	95 ± 4.9%	0 ± 0%	5 ± 4.9%	88.5 ± 7.6%	0.8 ± 1.8%	10.8 ± 8.2%
*Bupleurum rotundifolium* (*n* = 50)									
	A7	wild	20	37.1 ± 7.6%	54.2 ± 6.8%	8.7 ± 5%	0.3 ± 0.7%	91.2 ± 4.3%	8.5 ± 4.4%
	D1	cons.	20	86.9 ± 4.8%	3.9 ± 1.9%	9.2 ± 4.2%	78 ± 6.2%	13.4 ± 4.7%	8.6 ± 4.5%
	G8	cult.	20	57.9 ± 5.7%	10.5 ± 4%	31.6 ± 5.3%	10 ± 5.9%	57.2 ± 10.9%	32.8 ± 9.8%
*Cota tinctoria* (*n* = 200)									
	A8	wild	20	50.6 ± 6.1%	12.9 ± 3.9%	36.6 ± 8.3%	58.4 ± 6.9%	5.3 ± 1.8%	36.3 ± 7.4%
	A2	cult.	20	83 ± 4%	11.1 ± 3.5%	5.9 ± 2.3%	93.1 ± 2.4%	0.7 ± 0.8%	6.3 ± 2.4%
	G8	cult.	20	95.3 ± 1.8%	1.4 ± 0.7%	3.3 ± 1.8%	96.7 ± 1.4%	0.4 ± 0.4%	2.9 ± 1.4%
*Legousia speculum-veneris* (*n* = 50)									
	A3	wild	20	44.4 ± 12.6%	52.7 ± 12.4%	2.9 ± 2.1%	6 ± 4.9%	92.8 ± 5%	1.2 ± 1.2%
	A7	wild	20	14.1 ± 5.3%	83 ± 5.6%	2.9 ± 2.6%	31.8 ± 8.4%	66.9 ± 9%	1.3 ± 1.8%
	G3	wild	20	8.3 ± 6%	88.5 ± 7.7%	3.2 ± 5.2%	10.9 ± 4.9%	88.1 ± 4.8%	1 ± 1.4%
	D1	cons.	20	17.9 ± 6.7%	55.8 ± 8.8%	26.3 ± 8.9%	25.6 ± 6%	52.4 ± 12.4%	22 ± 11%
	G8	cult.	20	87.6 ± 7.1%	5.5 ± 4.2%	6.9 ± 4.8%	76.8 ± 7.6%	15.7 ± 7.9%	7.5 ± 3.8%
*Petrorhagia prolifera* (*n* = 50)									
	A4	wild	17	1.5 ± 1.8%	95.1 ± 4.4%	3.4 ± 4.3%	96 ± 3.2%	0.1 ± 0.5%	3.9 ± 3.4%
	A6	wild	9	42 ± 7.1%	54.4 ± 7.7%	3.6 ± 3.3%	98.2 ± 2.7%	0 ± 0%	1.8 ± 2.7%
	G4	wild	20	30.2 ± 7.3%	54.1 ± 6.6%	15.7 ± 6.5%	85.2 ± 5%	0.3 ± 0.7%	14.5 ± 5.1%
	G5	wild	12	21.8 ± 7.1%	11.8 ± 3.9%	66.3 ± 6.1%	38.8 ± 12.4%	0.2 ± 0.6%	61 ± 12.4%
	G8	cult.	20	53.8 ± 17.9%	41.6 ± 17.6%	4.6 ± 2.2%	96.6 ± 2.4%	0 ± 0%	3.4 ± 2.4%
*Ranunculus arvensis* (*n* = 20)									
	A8	wild	17	40.9 ± 11.5%	57.9 ± 11.6%	1.2 ± 2.8%	0.3 ± 1.2%	99.1 ± 2%	0.6 ± 1.7%
	G6	wild	20	29 ± 11.5%	61 ± 13.5%	10 ± 8.4%	0 ± 0%	94.5 ± 4.6%	5.5 ± 4.6%
	G7	wild	20	11 ± 8.4%	83.8 ± 8.9%	5.3 ± 4.4%	0 ± 0%	97 ± 3.8%	3 ± 3.8%
	G1	cons.	20	19.6 ± 12.1%	75.4 ± 13.2%	5 ± 5.2%	0 ± 0%	94.2 ± 5.1%	5.8 ± 5.1%
	G2	cult.	12	17.3 ± 8.8%	73 ± 7.7%	9.8 ± 8.7%	0.8 ± 2.4%	87.5 ± 8%	11.8 ± 7.3%
	G8	cult.	19	2.6 ± 3.1%	95 ± 5.3%	2.4 ± 3.5%	0 ± 0%	97.6 ± 3.5%	2.4 ± 3.5%
*Scandix pecten-veneris* (*n* = 20)									
	A7	wild	5	65 ± 7.9%	34 ± 8.2%	1 ± 2.2%	56 ± 14.7%	41 ± 16.4%	3 ± 2.7%
	A1	cons.	20	95.7 ± 3.5%	2.1 ± 3.9%	2.1 ± 2.7%	92.9 ± 3.9%	0.7 ± 1.9%	6.4 ± 2.4%
	G1	cons.	7	28 ± 10.8%	58.3 ± 10%	13.8 ± 7.9%	9.3 ± 7.1%	79.3 ± 11.6%	11.5 ± 6.7%
	G2	cult.	20	58.8 ± 8.3%	30.5 ± 10.9%	10.8 ± 6.5%	29.5 ± 8.3%	61 ± 11.2%	9.5 ± 7.1%
	G8	cult.	20	46.3 ± 10.6%	45.3 ± 11.4%	8.5 ± 8.3%	32.3 ± 13%	60.5 ± 14.7%	7.3 ± 6%
*Silene noctiflora* (*n* = 100)									
	A3	wild	20	86.4 ± 17.7%	13.4 ± 17.5%	0.3 ± 0.7%	72.8 ± 26.8%	27.1 ± 26.7%	0.2 ± 0.4%
	A5	cons.	9	79.4 ± 19.8%	14.4 ± 19.6%	6.1 ± 1.5%	71.3 ± 11.3%	23 ± 11.2%	5.7 ± 4.3%
	A2	cult.	20	82.8 ± 14.1%	17.2 ± 14%	0.1 ± 0.4%	72.1 ± 17.9%	27.9 ± 17.9%	0.1 ± 0.2%
	G8	cult.	20	65.5 ± 8%	25.2 ± 7.7%	9.4 ± 3.3%	55.1 ± 10.3%	37.9 ± 10.2%	7.1 ± 3.1%

**Table A2:** Mean ($\bar{x}$) percentage and standard deviation of (i) germinated and (ii) dormant seeds, and (iii) GRI for all populations of each species, sorted by origin. Germination percentages are given for the stratification and incubation phase, as well as post experimental phase (post phase) which is included into dormancy percentage. Origins are given as wild, conservation (cons.) and cultivated (cult.). Values were calculated based on viable seeds and are given as average across all replicates (r). Sample sizes (*n*) are given as number of seeds per Petri dish.

				Stratified Treatment					Incubated Treatment			
Species	Population	Origin	r	$\bar{x}$ Germination Strat. Phase	$\bar{x}$ Germination Inc. Phase	$\bar{x}$ Germination Post Phase	$\bar{x}$ Dormancy	$\bar{x}$ GRI	$\bar{x}$ Germination Inc. Phase	$\bar{x}$ Germination Post Phase	$\bar{x}$ Dormancy	$\bar{x}$ GRI
*Agrostemma githago* (*n* = 20)												
	A7	wild	5	100 ± 0%	100 ± 0%	0 ± 0%	0 ± 0%	18.9 ± 0.7%/d	100 ± 0%	0 ± 0%	0 ± 0%	48.8 ± 1%/d
	A9	wild	20	100 ± 0%	100 ± 0%	0 ± 0%	0 ± 0%	17.2 ± 0.6%/d	99.7 ± 1.2%	0 ± 0%	0.3 ± 1.2%	39.6 ± 2.3%/d
	A1	cons.	20	99.5 ± 1.6%	100 ± 0%	0 ± 0%	0 ± 0%	21.2 ± 0.9%/d	100 ± 0%	0 ± 0%	0 ± 0%	48.9 ± 1.2%/d
	A2	cult.	20	100 ± 0%	100 ± 0%	0 ± 0%	0 ± 0%	28.2 ± 1.1%/d	100 ± 0%	0 ± 0%	0 ± 0%	49.9 ± 0.3%/d
	D2	cult.	20	100 ± 0%	100 ± 0%	0 ± 0%	0 ± 0%	23.4 ± 0.9%/d	100 ± 0%	0 ± 0%	0 ± 0%	49.5 ± 0.9%/d
	D8	cult.	20	99 ± 2.6%	100 ± 0%	0 ± 0%	0 ± 0%	17.8 ± 1%/d	99.2 ± 1.9%	0.5 ± 1.6%	0.8 ± 1.9%	43.4 ± 3.1%/d
*Bupleurum rotundifolium* (*n* = 50)												
	A7	wild	20	0 ± 0%	40.5 ± 7.5%	0.1 ± 0.5%	59.5 ± 7.5%	1.6 ± 0.3%/d	0.3 ± 0.8%	0 ± 0%	99.7 ± 0.8%	0 ± 0%/d
	D1	cons.	20	62.1 ± 6.4%	95.7 ± 2.2%	0 ± 0%	4.3 ± 2.2%	4.4 ± 0.1%/d	85.3 ± 5.3%	1.3 ± 1.5%	14.7 ± 5.3%	7.7 ± 0.5%/d
	D8	cult.	20	64 ± 7.5%	84.7 ± 5.6%	0 ± 0%	15.3 ± 5.6%	4 ± 0.2%/d	15.1 ± 8.4%	0 ± 0%	84.9 ± 8.4%	1.5 ± 0.9%/d
*Cota tinctoria* (*n* = 200)												
	A8	wild	20	52.9 ± 4.7%	79.9 ± 4.6%	0.7 ± 0.6%	20.1 ± 4.6%	5.9 ± 0.4%/d	91.7 ± 2.7%	1 ± 0.9%	8.3 ± 2.7%	16.7 ± 1%/d
	A2	cult.	20	57.5 ± 3.4%	88.2 ± 3.7%	0.1 ± 0.3%	11.8 ± 3.7%	6.6 ± 0.3%/d	99.3 ± 0.8%	0.1 ± 0.2%	0.7 ± 0.8%	21.5 ± 1.4%/d
	D8	cult.	20	78.3 ± 3%	98.5 ± 0.7%	0.1 ± 0.3%	1.5 ± 0.7%	9.2 ± 0.3%/d	99.6 ± 0.5%	0.1 ± 0.3%	0.4 ± 0.5%	28.6 ± 1.1%/d
*Legousia speculum-veneris* (*n* = 50)												
	A3	wild	20	43.3 ± 12.3%	45.7 ± 12.8%	0 ± 0%	54.3 ± 12.8%	2.4 ± 0.7%/d	6.1 ± 4.9%	0.4 ± 1.1%	93.9 ± 4.9%	0.9 ± 0.8%/d
	A7	wild	20	2.1 ± 3%	14.5 ± 5.4%	4.9 ± 3.4%	85.5 ± 5.4%	0.5 ± 0.2%/d	32.3 ± 8.7%	3 ± 3.1%	67.7 ± 8.7%	4.6 ± 1.3%/d
	D3	wild	20	2.8 ± 3.3%	8.6 ± 6.2%	0.6 ± 1%	91.4 ± 6.2%	0.4 ± 0.3%/d	11 ± 4.9%	0.3 ± 1%	89 ± 4.9%	1.7 ± 0.8%/d
	D1	cons.	20	4.3 ± 5.2%	24.2 ± 8.2%	2.2 ± 3.5%	75.8 ± 8.2%	0.9 ± 0.4%/d	33.7 ± 10.8%	2.3 ± 2.8%	66.3 ± 10.8%	4.7 ± 1.8%/d
	D8	cult.	20	81.3 ± 7.8%	94 ± 4.8%	0.5 ± 1.2%	6 ± 4.8%	4.7 ± 0.3%/d	83.1 ± 8.2%	0.4 ± 0.9%	16.9 ± 8.2%	13.8 ± 1.4%/d
*Petrorhagia prolifera* (*n* = 50)												
	A4	wild	17	1.3 ± 1.9%	1.6 ± 1.9%	0 ± 0%	98.4 ± 1.9%	0.1 ± 0.1%/d	99.9 ± 0.5%	0 ± 0%	0.1 ± 0.5%	23.6 ± 1%/d
	A6	wild	9	43.4 ± 7.7%	43.6 ± 7.5%	0 ± 0%	56.4 ± 7.5%	4 ± 0.7%/d	100 ± 0%	0 ± 0%	0 ± 0%	31.8 ± 2.4%/d
	D4	wild	20	24.2 ± 7.2%	35.7 ± 7.5%	0.3 ± 0.8%	64.3 ± 7.5%	3.2 ± 0.8%/d	99.7 ± 0.8%	0.2 ± 0.7%	0.3 ± 0.8%	26.9 ± 1.1%/d
	D5	wild	12	26.4 ± 10.6%	63.6 ± 16.5%	0.6 ± 1.9%	36.4 ± 16.5%	4.4 ± 1.5%/d	99.6 ± 1.3%	0.4 ± 1.3%	0.4 ± 1.3%	29.7 ± 2.4%/d
	D8	cult.	20	25 ± 6.5%	56.3 ± 18.6%	1 ± 1.9%	43.7 ± 18.6%	3 ± 0.7%/d	100 ± 0%	0 ± 0%	0 ± 0%	28.8 ± 1%/d
*Ranunculus arvensis* (*n* = 20)												
	A8	wild	17	0.3 ± 1.2%	41.4 ± 11.6%	6.2 ± 7.2%	58.6 ± 11.6%	1.5 ± 0.4%/d	0.3 ± 1.2%	2.4 ± 3.7%	99.7 ± 1.2%	0 ± 0.1%/d
	D6	wild	20	0 ± 0%	32.4 ± 13.2%	11.2 ± 8.6%	67.6 ± 13.2%	0.9 ± 0.4%/d	0 ± 0%	0 ± 0%	100 ± 0%	0 ± 0%/d
	D7	wild	20	0 ± 0%	11.6 ± 8.7%	8.4 ± 7.4%	88.4 ± 8.7%	0.4 ± 0.3%/d	0 ± 0%	0 ± 0%	100 ± 0%	0 ± 0%/d
	D1	cons.	20	4.4 ± 3.9%	20.7 ± 13%	4.8 ± 5.1%	79.3 ± 13%	0.7 ± 0.5%/d	0 ± 0%	0 ± 0%	100 ± 0%	0 ± 0%/d
	D2	cult.	12	2.1 ± 3.2%	18.7 ± 8.7%	7.9 ± 6.7%	81.3 ± 8.7%	0.6 ± 0.3%/d	0.9 ± 2.8%	6.7 ± 7%	99.1 ± 2.8%	0.1 ± 0.3%/d
	D8	cult.	19	0 ± 0%	2.7 ± 3.2%	1.1 ± 3.2%	97.3 ± 3.2%	0.1 ± 0.1%/d	0 ± 0%	0.3 ± 1.2%	100 ± 0%	0 ± 0%/d
*Scandix pecten-veneris* (*n* = 20)												
	A7	wild	5	57.7 ± 8.6%	65.7 ± 8.1%	0 ± 0%	34.3 ± 8.1%	4 ± 0.5%/d	57.9 ± 15.8%	0 ± 0%	42.1 ± 15.8%	8.7 ± 2.5%/d
	A1	cons.	20	54.7 ± 12.8%	97.9 ± 3.9%	0 ± 0%	2.1 ± 3.9%	4.7 ± 0.3%/d	99.2 ± 2.1%	0 ± 0%	0.8 ± 2.1%	13.8 ± 0.7%/d
	D1	cons.	7	10.2 ± 7.8%	32.2 ± 11.9%	0.9 ± 2.1%	67.8 ± 11.9%	1.3 ± 0.5%/d	10.8 ± 9%	5.1 ± 6.1%	89.2 ± 9%	0.8 ± 0.7%/d
	D2	cult.	20	49.1 ± 10.1%	66.2 ± 10.6%	1.1 ± 2.3%	33.8 ± 10.6%	3.5 ± 0.7%/d	32.8 ± 9.8%	3.3 ± 3.7%	67.2 ± 9.8%	3.3 ± 1.2%/d
	D8	cult.	20	20.3 ± 11.4%	50.6 ± 11.2%	1.3 ± 2.9%	49.4 ± 11.2%	2.2 ± 0.6%/d	35 ± 14.2%	1.9 ± 2.7%	65 ± 14.2%	3.3 ± 1.4%/d
*Silene noctiflora* (*n* = 100)												
	A3	wild	20	0 ± 0%	86.6 ± 17.7%	3.1 ± 4.3%	13.4 ± 17.7%	2.5 ± 0.7%/d	72.9 ± 26.8%	5.9 ± 4.8%	27.1 ± 26.8%	6.7 ± 3.3%/d
	A5	cons.	9	0 ± 0%	84.6 ± 20.9%	4.9 ± 5.7%	15.4 ± 20.9%	2.4 ± 0.6%/d	75.7 ± 11.4%	4.1 ± 3.9%	24.3 ± 11.4%	5.5 ± 0.9%/d
	A2	cult.	20	0 ± 0%	82.8 ± 14%	11.2 ± 9%	17.2 ± 14%	2.5 ± 0.5%/d	72.1 ± 17.9%	10.6 ± 9.3%	27.9 ± 17.9%	6.9 ± 2.4%/d
	D8	cult.	20	0 ± 0%	72.2 ± 8.3%	6.8 ± 4%	27.8 ± 8.3%	2.1 ± 0.3%/d	59.3 ± 10.9%	8.4 ± 6.5%	40.7 ± 10.9%	4.7 ± 1%/d

## References

[1] Baskin C.C., Baskin J.M. (2014). Seeds: Ecology, Biogeography, and Evolution of Dormancy and Germination.

[2] Kildisheva O.A., Dixon K.W., Silveira F.A.O., Chapman T., Di Sacco A., Mondoni A., Turner S.R., Cross A.T. (2020). Dormancy and germination: Making every seed count in restoration. Restor. Ecol..

[3] Penfield S. (2017). Seed dormancy and germination. Curr. Biol..

[4] Penfield S., MacGregor D.R. (2017). Effects of environmental variation during seed production on seed dormancy and germination. J. Exp. Bot..

[5] Nonogaki H. (2014). Seed dormancy and germination-emerging mechanisms and new hypotheses. Front. Plant Sci..

[6] Baskin C.C., Baskin J.M. (2004). Germinating Seeds of Wildflowers, an Ecological Perspective. HortTechnology.

[7] Thompson K., Bakker J.P., Bekker R.M., Hodgson J.G. (1998). Ecological correlates of seed persistence in soil in the north-west European flora. J. Ecol..

[8] Meyer S., Leuschner C. (2015). 100 Äcker Für Die Vielfalt.

[9] Willcox G. (2012). Searching for the origins of arable weeds in the Near East. Veget. Hist. Archaebot..

[10] Rösch M. (1998). The history of crops and crop weeds in south-western Germany from the Neolithic period to modern times, as shown by archaeobotanical evidence. Veget. Hist. Archaebot..

[11] Marshall E.J.P., Brown V.K., Boatman N.D., Lutman P.J.W., Squire G.R., Ward L.K. (2003). The role of weeds in supporting biological diversity within crop fields. Weed Res..

[12] Smith B.M., Aebischer N.J., Ewald J., Moreby S., Potter C., Holland J.M. (2020). The Potential of Arable Weeds to Reverse Invertebrate Declines and Associated Ecosystem Services in Cereal Crops. Front. Sustain. Food Syst..

[13] Schlaepfer D.R., Braschler B., Rusterholz H.-P., Baur B. (2018). Genetic effects of anthropogenic habitat fragmentation on remnant animal and plant populations: A meta-analysis. Ecosphere.

[14] Albrecht H., Cambecèdes J., Lang M., Wagner M. (2016). Management options for the conservation of rare arable plants in Europe. Bot. Lett..

[15] Gerhards R., Dieterich M., Schumacher M. (2013). Rückgang von Ackerunkräutern in Baden-Württemberg-ein Vergleich von vegetationskundlichen Erhebungen in den Jahren 1948/49, 1975–1978 und 2011 im Raum Mehrstetten-Empfehlungen für Landwirtschaft und Naturschutz. Gesunde Pflanz..

[16] Meyer S., Wesche K., Krause B., Leuschner C. (2013). Dramatic losses of specialist arable plants in Central Germany since the 1950s/60s—A cross-regional analysis. Divers. Distrib..

[17] Brütting C., Wesche K., Meyer S., Hensen I. (2012). Genetic diversity of six arable plants in relation to their Red List status. Biodivers. Conserv..

[18] Storkey J., Meyer S., Still K.S., Leuschner C. (2012). The impact of agricultural intensification and land-use change on the European arable flora. Proc. Biol. Sci..

[19] Fried G., Petit S., Dessaint F., Reboud X. (2009). Arable weed decline in Northern France: Crop edges as refugia for weed conservation?. Biol. Conserv..

[20] Stoate C., Boatman N.D., Borralho R.J., Carvalho C.R., de Snoo G.R., Eden P. (2001). Ecological impacts of arable intensification in Europe. J. Environ. Manag..

[21] Lang M., Kollmann J., Prestele J., Wiesinger K., Albrecht H. (2021). Reintroduction of rare arable plants in extensively managed fields: Effects of crop type, sowing density and soil tillage. Agric. Ecosyst. Environ..

[22] Lang M., Albrecht H., Kollmann J., Himmler D. (2021). Ackerwildkräuter Für Bayerns Kulturlandschaft: Produktionsintegrierte Förderung Seltener und Gefährdeter Ackerwildkrautarten.

[23] Muchow T., Fortmann L. (2019). Concepts for the preservation of endangered arable wildflowers on the scale of natural regions: Experience from the pilot project ‘Weeds grow tall-not at all!. Nat. Und Landsch.-Z. Für Naturschutz Und Landschaftspflege.

[24] Epperlein L.R., Prestele J.W., Albrecht H., Kollmann J. (2014). Reintroduction of a rare arable weed: Competition effects on weed fitness and crop yield. Agric. Ecosyst. Environ..

[25] Kohler F., Vandenberghe C., Imstepf R., Gillet F. (2011). Restoration of Threatened Arable Weed Communities in Abandoned Mountainous Crop Fields. Restor. Ecol..

[26] Pedrini S., Gibson-Roy P., Trivedi C., Gálvez-Ramírez C., Hardwick K., Shaw N., Frischie S., Laverack G., Dixon K. (2020). Collection and production of native seeds for ecological restoration. Restor. Ecol..

[27] Nevill P.G., Cross A.T., Dixon K.W. (2018). Ethical seed sourcing is a key issue in meeting global restoration targets. Curr. Biol..

[28] Abeli T., Dalrymple S., Godefroid S., Mondoni A., Müller J.V., Rossi G., Orsenigo S. (2020). Ex situ collections and their potential for the restoration of extinct plants. Conserv. Biol..

[29] Cochrane J.A., Crawford A.D., Monks L.T. (2007). The significance of ex situ seed conservation to reintroduction of threatened plants. Aust. J. Bot..

[30] Guerrant E.O., Havens K., Maunder M., Guerrant E.O., Havens K., Maunder M., Raven P.H. (2004). Ex Situ Plant Conservation: Supporting Species Survival in the Wild.

[31] D’Agostino M., Abeli T. (2025). Biological flora of Central Europe: *Centaurea cyanus* L. Perspect. Plant Ecol. Evol. Syst..

[32] Ortega-Marcos J., Hevia V., García-Nieto A.P., González J.A. (2022). Installing Flower Strips to Promote Pollinators in Simplified Agricultural Landscapes: Comprehensive Viability Assessment in Sunflower Fields. Land.

[33] Uyttenbroeck R., Hatt S., Paul A., Boeraeve F., Piqueray J., Francis F., Danthine S., Frédérich M., Dufrêne M., Bodson B. (2016). Pros and cons of flowers strips for farmers. A review. Biotechnol. Agron. Soc. Environ..

[34] Haaland C., Naisbit R.E., Bersier L.-F. (2011). Sown wildflower strips for insect conservation: A review. Insect Conserv. Divers..

[35] Nagel R., Durka W., Bossdorf O., Bucharova A. (2019). Rapid evolution in native plants cultivated for ecological restoration: Not a general pattern. Plant Biol..

[36] Aavik T., Bosshard D., Edwards P., Holderegger R., Billeter R. (2014). Genetische Vielfalt in Wildpflanzen-Samenmischungen. Agrar. Schweiz.

[37] Keller M., Kollmann J., Edwards P.J. (2000). Genetic introgression from distant provenances reduces fitness in local weed populations. J. Appl. Ecol..

[38] Pedrini S., Dixon K.W. (2020). International principles and standards for native seeds in ecological restoration. Restor. Ecol..

[39] (2010). Commission Directive 2010/60/EU of 30 August 2010 Providing for Certain Derogations for Marketing of Fodder plant Seed Mixtures Intended for Use in the Preservation of the Natural Environment Text with EEA Relevance.

[40] Sandner T.M., Gemeinholzer B., Lemmer J., Matthies D., Ensslin A. (2022). Continuous inbreeding affects genetic variation, phenology, and reproductive strategy in ex situ cultivated Digitalis lutea. Am. J. Bot..

[41] Ensslin A., Tschöpe O., Burkart M., Joshi J. (2015). Fitness decline and adaptation to novel environments in ex situ plant collections: Current knowledge and future perspectives. Biol. Conserv..

[42] Brütting C., Hensen I., Wesche K. (2013). Ex situ cultivation affects genetic structure and diversity in arable plants. Plant Biol..

[43] Lauterbach D., Burkart M., Gemeinholzer B. (2012). Rapid genetic differentiation between ex situ and their in situ source populations: An example of the endangered *Silene otites* (Caryophyllaceae). Bot. J. Linn. Soc..

[44] Ensslin A., Sandner T.M., Godefroid S. (2023). Does the reduction of seed dormancy during ex situ cultivation affect the germination and establishment of plants reintroduced into the wild?. J. Appl. Ecol..

[45] Pizza R., Espeland E., Etterson J. (2021). Eight generations of native seed cultivation reduces plant fitness relative to the wild progenitor population. Evol. Appl..

[46] Ensslin A., van de Vyver A., Vanderborght T., Godefroid S. (2018). Ex situ cultivation entails high risk of seed dormancy loss on short-lived wild plant species. J. Appl. Ecol..

[47] Schröder R., Prasse R. (2013). From nursery into nature: A study on performance of cultivated varieties of native plants used in re-vegetation, their wild relatives and evolving wild × cultivar hybrids. Ecol. Eng..

[48] POWO Plants of the World Online. Facilitated by the Royal Botanic Gardens, Kew. Published on the Internet. https://powo.science.kew.org/.

[49] Oberdorfer E. (2021). Pflanzensoziologische Exkursionsflora: Für Deutschland und Angrenzende Gebiete.

[50] Sebald O., Seybold S., Philippi G. (1993). Die Farn- und Blütenpflanzen Baden-Württembergs: Band 1—Allgemeiner Teil, Spezieller Teil (Pteridophyta, Spermatophyta) Lycopodiaceae Bis Plumbaginaceae.

[51] Sebald O., Seybold S., Philippi G. (1992). Die Farn- und Blütenpflanzen Baden-Württembergs: Band 4—Spezieller Teil (Spermatophyta, Unterklasse Rosidae) Haloragaceae Bis Apiaceae).

[52] Sebald O., Seybold S., Philippi G. (1996). Die Farn- und Blütenpflanzen Baden-Württembergs: Band 5—Spezieller Teil (Spermatophyta, Unterklasse Asteridae) Buddlejaceae Bis Caprifoliaceae.

[53] Sebald O., Seybold S., Philippi G. (1996). Die Farn- und Blütenpflanzen Baden-Württembergs: Band 6—Spezieller Teil (Spermatophyta, Unterklasse Asteridae) Valerianaceae Bis Asteraceae.

[54] Mucina L., Grabherr G.G., Ellmauer T., Wallnhöfer S. (1993). Die Pflanzengesellschaften Österreichs: Teil I: Anthropogene Vegetation.

[55] European Native Seed Conservation Network (2009). Seed Collecting Manual for Wild Species.

[56] Lakon G. (1949). The topographical tetrazolium method for determining the germination capacity of seeds. Plant Physiol..

[57] Cottrell H.J. (1947). Tetrazolium salt as a seed germination indicator. Nature.

[58] Esechie H.A. (1994). Interaction of Salinity and Temperature on the Germination of Sorghum. J. Agron. Crop Sci..

[59] Kader M.A. (2005). A Comparison of Seed Germination Calculation A Comparison of Seed Germination Calculation Formulae and the Associated Interpretation of Resulting Data. J. Proc. R. Soc. N. S. Wales.

[60] Wilcox R. (2012). Introduction to Robust Estimation and Hypothesis Testing.

[61] R Core Team (2024). R: A Language and Environment for Statistical Computing.

[62] Posit Team (2025). RStudio: Integrated Development Environment for R.

[63] Kuhn M. (2008). Building Predictive Models in R Using the caret Package. J. Stat. Soft..

[64] Mair P., Wilcox R. (2020). Robust statistical methods in R using the WRS2 package. Behav. Res..

[65] Wickham H., François R., Henry L., Müller K., Vaughan D., Posit Software P.B. (2013). Dplyr: A Grammar of Data Manipulation.

[66] Wickham H., Vaughan D., Girlich M., Posit Software P.B. (2014). Tidyr: Tidy Messy Data.

[67] Wickham H., Averick M., Bryan J., Chang W., McGowan L., François R., Grolemund G., Hayes A., Henry L., Hester J. (2019). Welcome to the Tidyverse. J. Open Source Softw..

[68] Wickham H. (2016). Ggplot2: Elegant Graphics for Data Analysis.

[69] Society for Ecological Restoration, International Network for Seed Based Restoration and Royal Botanic Gardens Kew. Seed Information Database (SID). https://ser-sid.org/about.

[70] Hammer K., Hanelt P., Knüpffer H. (1982). Vorarbeiten zur monographischen Darstellung von Wildpflanzensortimenten: *Agrostemma* L. Die Kult..

[71] Zare A., Keshtkar E. (2025). Factors influencing germination and seedling emergence of corncockle (*Agrostemma githago*): Insights for weed management in agricultural systems. Acta Physiol. Plant.

[72] Brütting C., Meyer S., Kühne P., Hensen I., Wesche K. (2012). Spatial genetic structure and low diversity of the rare arable plant *Bupleurum rotundifolium* L. indicate fragmentation in Central Europe. Agric. Ecosyst. Environ..

[73] Yuan C., Liu Y., Wang Y., Nan T., Kang L., Li H., Zhan Z., Huang L. (2022). A comprehensive review of the phytochemistry, pharmacology, and saponin biosynthesis of the genus Bupleurum. Phytochem. Rev..

[74] McNeill J. (1980). THE BIOLOGY OF CANADIAN WEEDS.: 46. *Silene noctiflora* L. Can. J. Plant Sci..

[75] Qaderi M.M., Reid D.M. (2008). Combined Effects of Temperature and Carbon Dioxide on Plant Growth and Subsequent Seed Germinability of *Silene noctiflora*. Int. J. Plant Sci..

[76] Lang M., Prestele J., Fischer C., Kollmann J., Albrecht H. (2016). Reintroduction of rare arable plants by seed transfer. What are the optimal sowing rates?. Ecol. Evol..

[77] Baskin J.M., Baskin C.C. (2004). A classification system for seed dormancy. Seed Sci. Res..

[78] Cortinhas A., Ferreira T.C., Abreu M.M., Caperta A.D. (2021). Conservation of a Critically Endangered Endemic Halophyte of West Portugal: A Microcosm Assay to Assess the Potential of Soil Technology for Species Reintroduction. Front. Ecol. Evol..

[79] Żabicka J., Żabicki P., Słomka A., Sliwinska E., Jędrzejczyk-Korycińska M., Nowak T., Migdałek G., Kwiatkowska M., Kuta E. (2022). Re-introduction of an extinct population of Pulsatilla patens using different propagation techniques. Sci. Rep..

[80] Beckstead J., Meyer S.E., Allen P.S. (1996). Bromus tectorum seed germination: Between-population and between-year variation. Can. J. Bot..

[81] Tielbörger K., Petrů M. (2010). An experimental test for effects of the maternal environment on delayed germination. J. Ecol..

[82] Lu J.J., Tan D.Y., Baskin C.C., Baskin J.M. (2016). Effects of germination season on life history traits and on transgenerational plasticity in seed dormancy in a cold desert annual. Sci. Rep..

[83] Steadman K.J., Ellery A.J., Chapman R., Moore A., Turner N.C. (2004). Maturation temperature and rainfall influence seed dormancy characteristics of annual ryegrass (*Lolium rigidum*). Aust. J. Agric. Res..

[84] Matzrafi M., Osipitan O.A., Ohadi S., Mesgaran M.B. (2020). Under pressure: Maternal effects promote drought tolerance in progeny seed of Palmer amaranth (*Amaranthus palmeri*). Weed Sci..

[85] Chen D., Yuan Z., Wei Z., Hu X. (2022). Effect of maternal environment on seed germination and seed yield components of *Thlaspi arvense*. Ind. Crops Prod..

[86] Baskin C.C., Chesson P.L., Baskin J.M. (1993). Annual Seed Dormancy Cycles in Two Desert Winter Annuals. J. Ecol..

[87] Ensslin A., Godefroid S. (2020). Ex situ cultivation impacts on plant traits and drought stress response in a multi-species experiment. Biol. Conserv..

[88] Qu L., Wang X., Chen Y., Scalzo R., Widrlechner M.P., Davis J.M., Hancock J.F. (2005). Commercial Seed Lots Exhibit Reduced Seed Dormancy in Comparison to Wild Seed Lots of *Echinacea purpurea*. HortScience.

[89] Rauschkolb R., Szczeparska L., Kehl A., Bossdorf O., Scheepens J.F. (2019). Plant populations of three threatened species experience rapid evolution under ex situ cultivation. Biodivers. Conserv..

[90] Schröder R., Prasse R. (2013). Cultivation and Hybridization Alter the Germination Behavior of Native Plants Used in Revegetation and Restoration. Restor. Ecol..

[91] Lozada-Gobilard S., Pánková H., Zhu J., Stojanova B., Münzbergová Z. (2020). Potential risk of interspecific hybridization in ex situ collections. J. Nat. Conserv..

[92] Kulpa S.M., Leger E.A. (2013). Strong natural selection during plant restoration favors an unexpected suite of plant traits. Evol. Appl..

[93] Schröder R., Graf M.D., Jochum J., Rode G., Schemmel J., Thimm I. (2013). Testing the Effects of a Regionalized Seed Production on the Germination Behavior of Wild Plant Species. Ecol. Restor..

[94] Gao C., Bezemer T.M., van Bodegom P.M., Cornelissen H.C., van Logtestijn R., Liu X., Mancinelli R., van der Hagen H., Zhou M., Soudzilovskaia N.A. (2023). Plant community responses to alterations in soil abiotic and biotic conditions are decoupled for above- and below-ground traits. J. Ecol..

[95] Niklas K.J., Telewski F.W. (2022). Environmental-biomechanical reciprocity and the evolution of plant material properties. J. Exp. Bot..

[96] Müller C.M., Huwe B., Wissemann V., Joshi J., Gemeinholzer B. (2017). Conservation genetic assessment of four plant species in a small replica of a steppe ecosystem > 30 years after establishment. Biodivers. Conserv..

[97] Frankham R. (2008). Genetic adaptation to captivity in species conservation programs. Mol. Ecol..

[98] GeoSphere Austria (2020). SPARTACUS.

[99] Landesanstalt Für Umwelt Baden-Württemberg Klimaatlas: Klima der Vergangenheit. https://www.klimaatlas-bw.de/klima-vergangenheit.

[100] Harel D., Holzapfel C., Sternberg M. (2011). Seed mass and dormancy of annual plant populations and communities decreases with aridity and rainfall predictability. Basic Appl. Ecol..

[101] Liu Y., Barot S., El-Kassaby Y.A., Loeuille N. (2016). Impact of temperature shifts on the joint evolution of seed dormancy and size. Ecol. Evol..

[102] Klupczyńska E.A., Pawłowski T.A. (2021). Regulation of Seed Dormancy and Germination Mechanisms in a Changing Environment. Int. J. Mol. Sci..

[103] Brancaleoni L., Gerdol R., Abeli T., Corli A., Rossi G., Orsenigo S. (2018). Nursery pre-treatment positively affects reintroduced plant performance via plant pre-conditioning, but not via maternal effects. Aquat. Conserv. Mar. Freshw. Ecosyst..

[104] Maleki K., Vandelook F., Saatkamp A., Maleki K., Heshmati S., Soltani E. (2025). Global Patterns in the Evolutionary Relations Between Seed Mass and Germination Traits. Ecol. Evol..

[105] Fenner M., Thompson K. (2005). The Ecology of Seeds.

[106] Thompson K., Bakker J.P., Bekker R.M. (1997). The Soil Seed Banks of North West Europe: Methodology, Density and Longevity.

[107] Bergmeier E., Meyer S., Pape F., Dierschke H., Härdtle W., Heinken T., Hölzel N., Remy D., Schwabe A., Tischew S. (2021). Arable Wildflower Vegetation of Calcareous Fields (Caucalidion): Plant Community of the Year 2022: (Ackerwildkraut-Vegetation der Kalkäcker (Caucalidion): Pflanzengesellschaft des Jahres 2022). Tuexenia.

[108] Hegi G. (1965). Illustrierte Flora von Mitteleuropa—Pteridophyta, Spermatophyta: Band III Teil 3 Angiosperma, Dicotyledones 1.

[109] Hegi G. (1975). Illustrierte Flora von Mitteleuropa—Dicotyledones: V. Band, 2. Teil Cactaceae—Cornaceae.

[110] Stoyanov S., Apostolova-Stoyanova N. (2024). Checklist of the flora in the Rusenski Lom River Valley (Northeast Bulgaria). Phytol. Balc..

[111] Basey A.C., Fant J.B., Kramer A.T. (2015). Producing native plant materials for restoration: 10 rules to collect and maintain genetic diversity. Nativ. Plants J..

[112] Burney D.A., Burney L.P. (2007). Paleoecology and “inter-situ” restoration on Kaua’i, Hawai’i. Front. Ecol. Environ..

[113] Burney D.A., Burney L.P. (2009). *Inter situ* conservation: Opening a “third front” in the battle to save rare Hawaiian plants. BGjournal.

[114] Volis S., Blecher M. (2010). Quasi in situ: A bridge between ex situ and in situ conservation of plants. Biodivers. Conserv..

